# From bench to bedside: an interdisciplinary journey through the gut-lung axis with insights into lung cancer and immunotherapy

**DOI:** 10.3389/fimmu.2024.1434804

**Published:** 2024-09-05

**Authors:** David Dora, Emőke Szőcs, Ádám Soós, Viktória Halasy, Csenge Somodi, Anna Mihucz, Melinda Rostás, Fruzsina Mógor, Zoltan Lohinai, Nándor Nagy

**Affiliations:** ^1^ Department of Anatomy, Histology and Embryology, Semmelweis University, Budapest, Hungary; ^2^ Translational Medicine Institute, Semmelweis University, Budapest, Hungary; ^3^ Department of Biochemistry and Molecular Biology, University of Debrecen, Debrecen, Hungary

**Keywords:** gut-lung axis, gut microbiome, mucosal immunity, gut permeability, lung cancer, immunotherapy

## Abstract

This comprehensive review undertakes a multidisciplinary exploration of the gut-lung axis, from the foundational aspects of anatomy, embryology, and histology, through the functional dynamics of pathophysiology, to implications for clinical science. The gut-lung axis, a bidirectional communication pathway, is central to understanding the interconnectedness of the gastrointestinal- and respiratory systems, both of which share embryological origins and engage in a continuous immunological crosstalk to maintain homeostasis and defend against external noxa. An essential component of this axis is the mucosa-associated lymphoid tissue system (MALT), which orchestrates immune responses across these distant sites. The review delves into the role of the gut microbiome in modulating these interactions, highlighting how microbial dysbiosis and increased gut permeability (“leaky gut”) can precipitate systemic inflammation and exacerbate respiratory conditions. Moreover, we thoroughly present the implication of the axis in oncological practice, particularly in lung cancer development and response to cancer immunotherapies. Our work seeks not only to synthesize current knowledge across the spectrum of science related to the gut-lung axis but also to inspire future interdisciplinary research that bridges gaps between basic science and clinical application. Our ultimate goal was to underscore the importance of a holistic understanding of the gut-lung axis, advocating for an integrated approach to unravel its complexities in human health and disease.

## Introduction

The exploration of the gut-lung axis, a bidirectional communication pathway between the gastrointestinal (GI) and respiratory systems, has expanded into a central area of research, shedding light on novel insights into human health and disease. This multi-layered interplay besets a spectrum of physiological, immunological, and microbial interactions, with implications for various pathological conditions. The gut-lung axis represents a complex linkage between two major organ systems, with shared embryological origins from the foregut (1). The mucosal immune systems of both, namely the gut-associated lymphoid tissue (GALT) and the bronchus-associated lymphoid tissue (BALT), all integral parts of the mucosa-associated lymphoid tissue (MALT) serve as integral components of this axis, orchestrating immune responses across these distal sites with a significant influence on systemic immunity (2–4).

The gut microbiome, a diverse assembly of microbes residing in the GI tract, plays a fundamental role in human physiology, extending beyond digestion to immune modulation and systemic health (5–8). This microbial-immune crosstalk is pivotal in maintaining respiratory health, with dysbiosis in the gut microbiota linked to altered immune responses and increased susceptibility to lung diseases. Increased gut permeability, colloquially known as ‘leaky gut’ and characterized by a compromised intestinal barrier, facilitates the translocation of microbial products into the systemic circulation (9, 10) and can trigger systemic inflammatory responses, potentially exacerbating respiratory pathologies (11). The resultant systemic immune activation, driven by microbial translocation, underlines the role of gut permeability in modulating the gut-lung axis dynamics (4).

The existence of the gut-lung axis signifies a flow of microbial and immunomodulatory signals and molecules. These mediator particles play a key role not only in local immune regulation but also in long-reaching immunomodulatory mechanisms. Through inhalation, sputum swallowing, or the mesenteric lymphatic system and the systemic circulation, intact bacteria, their particles, or metabolites can regulate inflammatory processes in both organ systems (12). The gut-lung axis has emerged as a significant factor in various diseases, including chronic respiratory conditions, GI disorders, and systemic inflammatory diseases (4, 13, 14). The gut disturbances in lung diseases, such as allergy, asthma, and chronic obstructive pulmonary disease (COPD) have been extensively studied, pointing out the conspicuous cross-talk between gut microbiota and the lungs (15–18). Its role in infectious diseases, notably in the context of COVID-19, has also highlighted its importance in disease pathogenesis and progression (19, 20).

In oncology, the gut-lung axis has garnered particular attention, especially in lung cancer development (21–23) and the response to cancer therapies, including immunotherapy. The gut microbiota’s influence on the efficacy and toxicity of cancer immunotherapies is increasingly recognized, with specific microbial compositions associated with improved therapeutic outcomes (24–29). Modulating the gut microbiota has been proposed as a strategy to enhance the efficacy of immunotherapies and mitigate adverse effects (30, 31). Lung cancer, one of the most prevalent and lethal cancers, may be influenced by the gut microbiota through mechanisms involving systemic inflammation, immune modulation, and the production of carcinogenic metabolites (32, 33), however, robust evidence of direct causality is still missing in some cases. The gut microbiome’s role in modulating the response to cancer therapies, including chemotherapy and immunotherapy, is currently among the most debated fields of studies (30, 34).

The essential role of the gut microbiome in the gut-lung axis, particularly in modulating immune responses and influencing disease pathogenesis, opened new avenues for therapeutic interventions. In the context of oncology, leveraging the gut-lung axis, especially in the field of lung cancer and cancer immunotherapy, offers promising prospects for advancing treatment strategies and improving patient outcomes. In this comprehensive review, we aim to present this physiological phenomenon through a broad range of disciplines, embracing the whole spectrum of biomedical science in the field. Starting with the embryonic development and microscopic morphology of the GI tract and the lungs, our goal was to deduce the inner workings of this system from its morpho-functional unit, directly extracting the translational relevance and clinical perspectives from basic science. We also aim to dissect intriguing topics such as the problem of gut permeability, gut dysbiosis-driven cancer formation in the lungs and immunotherapy efficacy, where we tried to exemplify the most intriguing biological phenomena within the gut-lung axis. In some of these fields robust scientific evidence is only in its emerging phase and most cited studies are exploratory in their nature, despite their strong clinical potential. Therefore, drawing clear clinical conclusions from our review requires prudence and cautious interpretation.

## Development and cytoarchitecture of the gut and the lungs: a comparison

### Common origin, different structures: development of the gut and the lungs

The GI tract and the respiratory system are organs of the amniote vertebrate body that have common developmental origins, form in a common embryonic body cavity, and possess similar histological structures. Both systems arise from the primitive gut tube, an embryonic structure containing an inner endodermally-derived epithelium surrounded by mesenchyme of splanchnic mesoderm origin (35). The primitive gut tube initially extends from the stomodeum to the cloaca, and it is subdivided into three basic parts: the foregut, midgut and hindgut.

### Morphogenesis

The epithelium forming the respiratory diverticulum is pinched off from the foregut endoderm of the 4-week-old human embryo and forms a parallel epithelial tube, anteriorly the trachea and posteriorly the esophagus, while the small- and large intestines develop from the midgut and hindgut portions, respectively. After the 5th week of gestation, the caudal end of the trachea dichotomously bifurcates and the left and right primary bronchial buds are formed, which continue to grow into the adjacent layer of pleural mesenchyme derived from the splanchnopleural mesoderm. The endoderm of the respiratory diverticulum gives rise to the epithelium and sero-mucous glands of the trachea, bronchi and bronchioles, as well as the pneumocytes lining of the alveolar ducts, while the splanchnic mesoderm contributes to the loose connective tissue, hyalin cartilage, and visceral smooth muscles of the lungs (36, 37). The same germ layers contribute to the formation of specific structures within the gut tube where the gut endoderm gives rise to the epithelium of the mucosa, the mucosal- and submucosal glands, while the mesoderm contributes to the lamina propria, muscularis mucosae, submucosal connective tissue, muscularis externa, and the outermost layers: the adventitia or serosa (38, 39).

### Molecular embryology

In addition to the common embryonic origin, a series of complex interactions between different cell types and signaling pathways are shared during the developmental biology processes regulating lung and gut development. Understanding the molecular processes that govern the lung and gut development is essential for identifying common congenital abnormalities and developing targeted interventions. The tissue interactions between the foregut epithelium and mesenchyme, along with signaling molecules such as fibroblast growth factors (FGFs) and bone morphogenetic proteins (BMPs) play crucial roles in trachea formation and branching morphogenesis of the lung buds (35, 40). FGFs induce the expression of lung-specific transcription factors, including NK2 homeobox 1 (previously TTF-1; NKX2–1, NKX2.1), which regulates the expression of surfactant genes (41). Wnt signaling has been identified as inducing fibronectin deposition and, consequently, regulate the determination of branch points. Similarly, the BMP4 is also regulating the branching lung buds, it is expressed predominantly in the lung epithelium and is increased at branch tips and acts as a lateral inhibitor of budding (42). Sonic hedgehog (SHH) signaling, on the other hand, helps establish the boundaries between the respiratory and esophageal regions (43). Studies using knockout mice have confirmed the significance of SHH mediated pathway in the specification of lung primordia and foregut development. SHH-null mutant mice exhibit similar characteristics to human foregut defects, such as esophageal atresia/stenosis, tracheoesophageal fistula, and anomalies in the trachea and lungs (44–46). In these mutants, the failure to develop the tracheoesophageal septum leads to incomplete separation of the two endodermal tubes, indicating the critical role of SHH in the proper development of the esophagus, trachea, and lungs. Once the respiratory fate is established, the ventral foregut endoderm forms two lung buds through evagination, which will eventually give rise to the bronchial tree and lung lobes.

Regarding the digestive part of the foregut, the endodermal epithelium gives rise to various organs, including the esophagus, stomach, intestines, and associated glands. The formation of these structures is regulated by a complex interaction of similar signaling molecules and transcription factors. For instance, SHH signaling is important for specifying the foregut region, while WNT and BMP signaling contribute to the patterning and differentiation of the digestive tract epithelium (47–49). As development progresses, the endodermal cells undergo further differentiation into specific cell types within each organ. In the respiratory tract, endodermal cells differentiate into ciliated cells, mucus-secreting cells, and pneumocytes involved in gas exchange. In the digestive tract, endodermal cells differentiate into absorptive enterocytes, mucus-secreting goblet cells, hormone-secreting endocrine cells, among others.

Notch signaling also plays a fundamental role in regulating lung and intestinal epithelial cell fate decisions and differentiation. In humans, the Notch signaling pathway consists of four different receptors (NOTCH1, NOTCH2, NOTCH3, and NOTCH4) and five canonical ligands (Jagged-1, Jagged-2, Delta-like 1 (DLL1), DLL3, and DLL4) (50). WNTs, Hhs, and BMPs have all been previously demonstrated to cooperate with NOTCH however, in contrast to these signaling processes, where secreted morphogens bind to their cognate receptors, in the case of NOTCH signaling pathways, transmembrane ligands expressed on one cell and activate transmembrane receptors on the adjacent cell (51). In the early lung bud, Notch signaling regulates epithelial progenitor cell maintenance, branching morphogenesis, and alveolar and airway epithelial cell differentiation. Experimental activation of Notch in mouse embryos inhibits differentiation of distal lung progenitors into alveolar cells (52, 53). In adult mice, activation of Notch signaling increases the number of airway mucous cells and decreases the number of ciliated cells to regulate the fine balance of the ciliated and mucous epithelial cell differentiation (53). In addition, during postnatal life Notch signaling is also required to restrict the differentiation of club cells, a non-ciliated secretory epithelial cells (54) into goblet cells (55, 56).

Similar to the developing lung, Notch signaling controls intestinal stem cell pool maintenance, determining the fate of Lrg5^+^ progenitor cells within the intestinal epithelium, and directing them toward either absorptive or secretory lineages in the intestinal crypt (57–59). High levels of Notch signaling promote the differentiation of progenitor cells into enterocytes, whereas inhibition of Notch signaling supports the differentiation into secretory cells, such as goblet cells (60). The Notch-mediated shift towards enterocyte differentiation explains why excessive mucus secretion, due to abnormal increase of goblet cells, is commonly observed after treatments with small molecule inhibitors of the γ-secretase protease complex (NOTCH inhibitors) (51, 61).

### Innervation and the neural crest

Trunk neural crest cells also play a crucial role in the development of sympathetic and sensory innervations to the lungs and the gut. Avian neural tube grafting experiments and studies using Wnt1;tdT transgenic mice have shown that neural crest cells (NCC) of vagal origin migrate from the foregut into the lung buds and differentiate into neurons and glia (62, 63). Studies on vagal NCC-derived enteric nervous system (ENS) formation in the GI tract have provided further insights into shared molecular mechanisms regulating the colonization of developing lung and intestine, such as transcription factors (SOX10, PHOX2b), growth factors (GDNF and Endothelin-3) and their cognate receptors, the RET and EdnrB respectively (48). The RET gene encoding a tyrosine-kinase receptor, plays a crucial role in the development of ENS, and mutations in this gene are associated with Hirschsprung disease (HSCR), a congenital disorder characterized by the absence of enteric ganglia in distal colorectum, leading to functional obstruction (64). RET has been also implicated in both NCC development in the lung and the neural pathway of respiratory carbon dioxide chemosensitivity. GDNF, which signals through a receptor complex including the RET tyrosine kinase and the GFRα1 co-receptors, is also a chemoattractant for NCCs in the lung. Experimental evidence has shown that GDNF expressed in the gut wall can attract neural crest cells expressing RET, and that GDNF-soaked beads transplanted into the mouse lung buds can attract RET-/Gfrα1^+^ neural crest cells and induce neuronal differentiation. A significant percentage of patients with congenital central hypoventilation syndrome (CCHS), a developmental disorder characterized by inadequate autonomic control of respiration and decreased sensitivity to hypoxia and hypercapnia also have Hirschsprung’s disease. Mutations in RET and PHOX2B genes have been found in patients with CCHS and HSCR, suggesting a common role for PHOX2B transcription factor and GDNF-RET- Gfrα1 signaling in congenital respiratory and neurointestinal disease conditions (65, 66). Another condition that can impact both the innervation of the gut and the lungs is multiple endocrine neoplasia type 2B (MEN 2B), a rare genetic syndrome characterized by thyroid cancer, pheochromocytomas and mucosal neuromas leading to neuropathy affecting both the respiratory and GI systems. Individuals may experience problems related to dysfunctional autonomic innervation, such as altered motility and respiratory difficulties. This syndrome is caused by mutations in the RET proto-oncogene, which plays a critical role in the development of cells derived from the neural crest, impacting both the ENS and parts of the autonomic nervous innervating the lungs. system (which influences lung function (67).

These findings of the gut-lung axis’s developmental origin highlight the shared molecular and structural bases of these vital systems. Originating from the primitive gut tube, both the GI and respiratory systems share embryonic origins, signaling pathways, and molecular regulators, indicating the importance of these early interactions in normal and pathological states. The engagement of signaling molecules like FGFs, BMPs, SHH and NOTCH receptors plays an important role in the morphogenesis and cellular differentiation within these systems. These signaling pathways not only dictate the structural formation of the trachea, lungs, and GI tract but also influence congenital conditions such as tracheoesophageal fistula and Hirschsprung disease. Additionally, the interaction between neural crest cells and these embryonic tissues highlights a significant overlap in the neurodevelopmental pathways influencing both respiratory and gastrointestinal functions.

## Mucosal cytoarchitecture of the gut and the lungs

The histological structure of the respiratory and GI tracts exhibits similarities as both pathways facilitate the passage of inhaled air or food, either actively or passively, while the epithelium interacts with, transports, and modifies the composition of the transported material. Consequently, the epithelium remains continuously exposed to the external environment, with a basic function of preserving barrier integrity and internal homeostasis. Their similarities originate in their common developmental origin at the level of organogenesis, signal transduction pathways, and microbiome formation (68–70).

### Epithelial cells

Extensive examination of the respiratory and digestive tracts - both histologically and at the molecular level - has been ongoing for numerous years. In both systems, the epithelial layer (lamina epithelialis) consists of a single-layer cylindrical epithelium, distinguished by its unique feature among other cylindrical epithelia: its cells exhibit glandular activity, hence termed as a secretory covering epithelium. In the airways, a pseudostratified columnar ciliated epithelium characterizes the luminal layer, comprising 15 distinct cell types grouped into 10 main clusters: ciliated cells, club cells, goblet cells, ionocytes, neuroendocrine cells, serous cells, mucous cells (in glands), pneumocytes type-I and II (in alveoli), and stem cells (71). Likewise, the epithelium of the small and large intestine is a monolayer of columnar epithelial cells, housing 7 primary cell types: enterocytes with microvilli, goblet cells, Paneth cells (predominantly in the small intestine), enteroendocrine cells, progenitor cells, transit amplifying (TA) cells, and stem cells. Intraepithelial lymphocytes intersperse among the intestinal epithelial cells in both systems (72). The guts and lungs share identical cell types with similar functions: for instance, goblet cells, with their footed cup shape and secretory vesicles, contribute to the production of a protective mucus layer. Additionally, tuft cells, rich in microvilli on their luminal surface exhibit chemosensory and immunomodulatory properties, found not only in the GI system but also in the respiratory and excretory apparatus (73). Neuroendocrine cells, acting as airway sensors in clusters of 3-20 cells within the mouse lung, play a pivotal role in immune response induction through neuropeptides (74) and neuronal responses (mediating neuroinhibition) (75) and bronchoconstriction (76), and vasodilation (70). Similarly, enteroendocrine cells, comprising 1% of the total intestinal epithelial cell population, regulate digestion, blood circulation, and absorption of nutrients while coordinating appetite through the secretion of approximately 20 bioactive hormones (77, 78).

These cell populations display varying distribution patterns across specific segments within both organ systems. The ciliated columnar epithelial cells and pneumocytes that line the airways are subdivided into proximal and distal sections. These cells facilitate the movement of the mucus layer with their cilia, whereas, in the alveoli, they undergo flattening (pneumocyte I) to serve as the primary site for gas exchange. Goblet cells, ionocytes, and neuroendocrine cells are predominantly found in the proximal airway, with minimal presence in the distal sections (71). Secretory club cells, largely abundant in the respiratory epithelium, constitute approximately 9% of the total epithelial cell population in the human lung, particularly in the distal segment of the bronchial tree (79). These cells also have a progenitor function in addition to the defense function against from toxins by secreting anti-inflammatory proteins (e.g. uteroglobin) from the respiratory system (80, 81). Pulmonary ionocytes primarily regulate luminal pH and are speculated to play a significant role in cystic fibrosis pathology, despite constituting only 1-2% of the epithelium (82).

**Figure 1:**
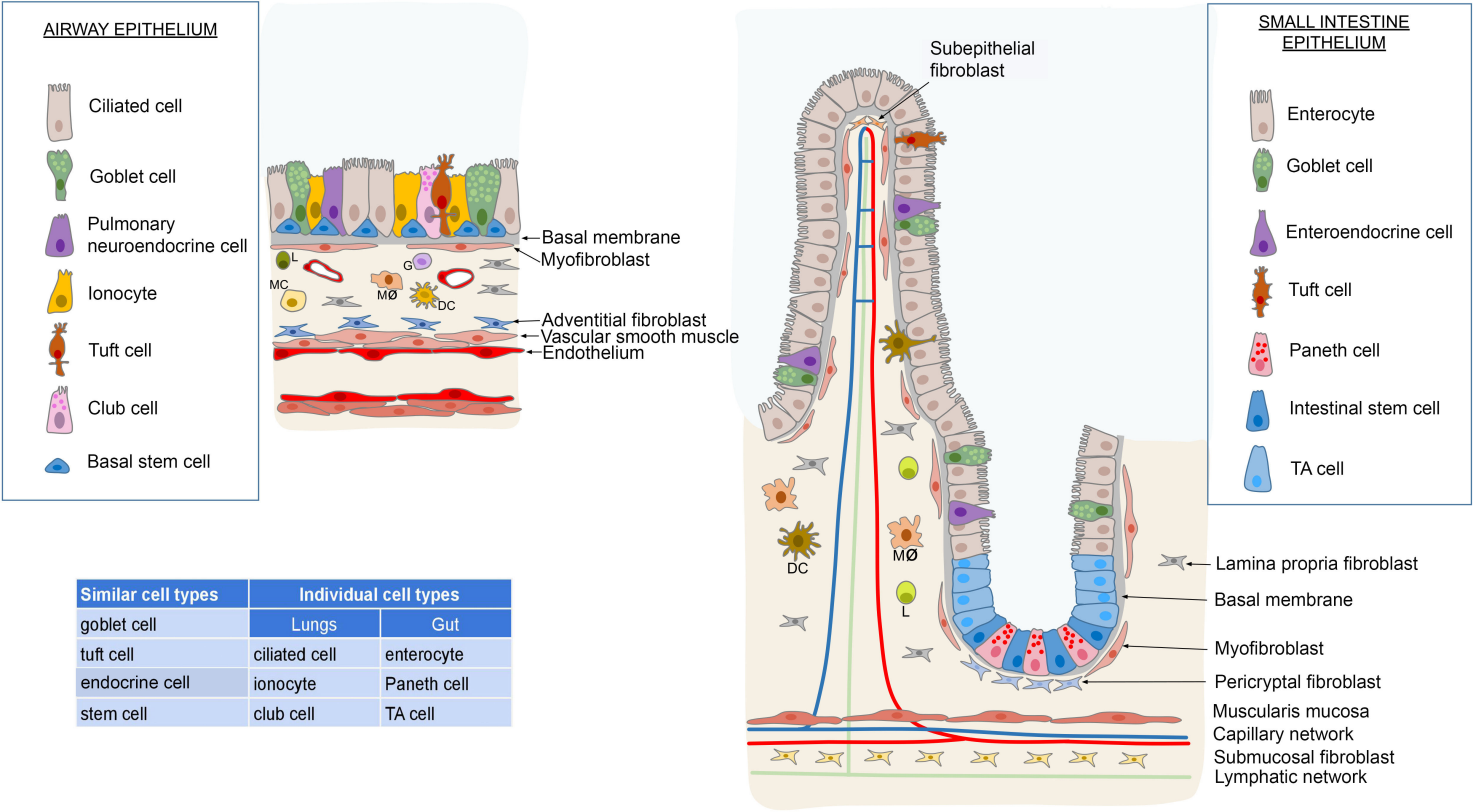
Comparison of the epithelial composition between the respiratory tract and the intestinal system reveals commonalities and distinctions. Both systems feature goblet cells, endocrine cells, stem cells and tuft cells interspersed among the cylindrical epithelial cells. However, unique cell types are present in each system; the respiratory system includes ciliated cells, club cells and ionocytes, while the intestinal system contains enterocytes, Paneth cells and transit-amplifying (TA) cells. In terms of stem cell distribution, the respiratory system’s stem cells are uniformly distributed along the basal membrane. In contrast, the small and large intestines house stem cells at the base of the Lieberkühn crypts. These crypts facilitate the replenishment of the intestinal epithelium through a population of TA cells. Beneath the basal membrane lies a layer rich in myofibroblasts, associated with lymphoreticular tissue in the lamina propria. This tissue harbors diverse immune cells such as lymphocytes (L), monocytes (MC), macrophages (Mø), dendritic cells (DC), and granulocytes (G), alongside specialized fibroblasts and glands. In the intestinal tract, this layer transitions into the submucosa, delineated by the lamina muscularis mucosae. Conversely, in the respiratory system, the lower boundary of the tunica epithelialis is demarcated by the tunica muscularis. Illustrations were made using MS PowerPoint and Adobe Illustrator. Images were compiled by Adobe Photoshop.

Transcriptome analysis of the ileum, jejunum, and colon also showed a different cell composition, with the intestinal absorptive enterocytes dominating the ileal epithelium by 70%, compared to only 14% in the colon and rectum, which are responsible for water retention and maintenance of the microbiome barrier (72). Interestingly, both the microbiota and pathogens exert an influence on the differentiation of intestinal stem cells through immune-dependent regulation according to the composition of the epithelium, and the overall physiology of the intestine. In pursuance of Liu’s study, the intricate network of molecular connections resolves the behavior of stem cells by synchronizing the activity of pathways implicated in microbial pattern recognition and the detection of epithelial damage (damage-associated molecular patterns). Due to this, while the microbiota promote the commitment of stem cells to the enterocyte lineage, pathogens stimulate the fate of enteroendocrine cells (83). Furthermore, the mycobiome affects the establishment of the appropriate stem cell niche especially during early postnatal development by influencing the differentiation of macrophages and mesenchymal cells to support the Paneth cell lineage (84). In contrast to enterocytes, the proportion of goblet cells is inversely distributed in the intestines, with a higher percentage in the colon (20%) producing protective mucus for the entire epithelial surface, compared to only 5% in the ileum. Enteroendocrine cells are found in low numbers throughout the intestinal tract, with an overabundance in the rectum (72). **Figure 1** demonstrates the epithelial cytoarchitecture of the gut- and pulmonary mucosa.

### Vasculature and connective tissue

The lamina propria of the respiratory tract hosts numerous capillaries exhibiting distinct molecular markers such as matrix composition, fenestrated morphology, and cell cycle characteristics, which differ from those surrounding the bronchi, indicative of their position in the pulmonary circulation. Additionally, fibroblasts within the subepithelial stroma display variations, with vascular adventitia encircling the alveoli demonstrating matrix biosynthesis, adhesion, and signal regulation functions (71). In the gut, a subepithelial layer of myofibroblasts lies beneath the basement membrane, comparable to the lung, accompanied by pericytes associated with capillaries and lymphatic vessels running along the axis of the villi (85). Composed of a cellular connective tissue affluent in mesenchymal stem cells and fibroblasts, particularly in the upper part of the villus, the lamina propria houses the majority of immune cells in the middle of the villus, facilitating interactions with the microbiome (69, 86, 87). At the border interfacing with the submucosa, the lamina muscularis mucosae encloses the lamina propria (88).

### Innervation

The autonomic nervous system’s afferent and efferent sympathetic and parasympathetic neurons provide innervation to the airways and lungs. Reflex regulation of autonomic function is facilitated by bronchopulmonary afferent fibers of the vagus nerve. The vagus nerve innervates the airways through parasympathetic and sensory fibers, organized into several subtypes, which are distributed throughout the bronchus, bronchioles, alveoli, accompanying the vasculature and lymphatics, and innervating the smooth muscle, epithelium, and neuroendocrine cells (89, 90). In the respiratory tract, comparable to the ENS, the cell bodies of extrinsic fibers of preganglionic neurons reside outside the respiratory apparatus, primarily in the medulla, mainly from the nucleus ambiguus, with a minor proportion originating from the dorsalis motor nucleus of the vagus nerve. Conversely, the cell bodies of postganglionic neurons (intrinsic pulmonary neurons) are organized into ganglia within the walls of the trachea and bronchi (91), which are of neural crest origin in both mice (63) and human embryos (62, 92). The sympathetic nerve cell bodies are found in the thoracic spinal cord intermedolateral nuclei, while the postganglionic neurons reside in the paravertebral sympathetic chain, originating from the stellate and thoracic segments. These neurons connect to intrapulmonary ganglia with fibers from the stellate and superior cervical ganglia. The respiratory (93) and GI systems are innervated by two sets of afferent nerves, with their cell bodies located in the nodose ganglion of the vagus nerve and the dorsal root ganglia, respectively (93, 94).

For the innervation of the intestinal nervous system, efferent preganglionic neurons in the thoracolumbar spinal cord form synapses with postganglionic neurons in the prevertebral (ggl. coeliac, superior and inferior mesenteric) and pelvic ganglia, which then innervate the intestines. Similarly to the respiratory system, parasympathetic preganglionic neurons arise from the dorsal motor nucleus of the vagus nerve and extend from the intermedolateral horn of the sacral spinal cord. These neurons connect with postganglionic neurons in the pelvic ganglia and submucosal and myenteric plexuses of the enteric nervous system (95, 96). The preganglionic axons of the vagus nerve innervate the intestinal tract up to the duodenum (97–99). Structural and functional similarities of the GI and respiratory tract stem from shared developmental pathways, signaling mechanisms, and microbiome influences. Each system has a variety of specialized cell types, such as mucus-secreting goblet cells and neuroendocrine cells that participate in immune and physiological regulation. The presence and function of these cells differ across the tracts, affecting mucus transport, gas exchange, and nutrient uptake. The role of the microbiota in these processes is significant, influencing stem cell differentiation into various cell lineages. Additionally, the surrounding structures, including the lamina propria and the autonomic nervous system innervation, are vital for the regulation and operation of these systems.

## The innate immune system of the gut and the lungs: development and cells of mucosal immunity

Mucosal surfaces are continuously exposed to high loads of antigens, therefore keeping the balance between immune response to pathogenic microorganisms and immune tolerance to commensal organisms is tightly controlled (100). Signals from various mucosal antigens are integrated in secondary lymphoid organs (SLOs), which serve as inductive sites for the immune system. Along the GI and respiratory tracts several specialized mucosa-associated lymphoid structures (MALT) are placed at strategic sites, which together with gut-draining mesenteric lymph nodes and bronchopulmonary lymph nodes orchestrate adequate immune responses. These structures develop at predefined sites during embryogenesis, independently of antigen signals and involve a series of cell-cell, cell-chemokine interactions (101).

### Formation of the gut-associated lymphoid tissue

Gut-associated lymphoid tissue (GALT) includes cryptopatches, isolated lymphoid follicles (ILFs), Peyer’s patches (PP), cecal patches and colonic patches that form a complex system along the GI tract, with several homeostatic and inflammatory functions (102, 103). PP development together with lymph node (LN) organogenesis has been extensively studied and relies on the initial clustering and interaction of lymphoid tissue inducer cells (LTi) with mesenchymal lymphoid tissue organizer (LTo) cells, that together with the involvement of homeostatic cytokines and chemokines drive the structural organization of SLOs throughout the embryo. LTi cells are fetal liver derived CD117^+^LTα1β2^+^CXCR5^+^IL-7Rα^+^RANKL^+^ subpopulation of type 3 innate lymphoid like cells (ILC3), dependent on the expression of RORγt (104, 105). Conditional knock-out of RORγt results in the absence of LTi cells and consequently LNs and PPs, which highlights the indispensible role of this cell type (106–108). Establisment of the PP anlagen starts with the recruitment of RET^+^ and LTβ^+^ lymphoid tissue initiatior cells (LTin), that interact with VCAM+ mesenchymal cells, expressing RET ligands (109, 110). This initial interaction results in the differentiation and activation of VCAM^+^ mesenchymal cells to LTo cells that express IL-7 and CXCL13 and recruit CXCR5^+^ LTi cells (111–113). Circulating lymphocytes are attracted and retained in the PP anlagen through the enhanced expression of cytokines (IL-7, RANK ligand), chemokines (CXCL12, CXCL13, CCL19, and CCL21), and adhesion molecules (VCAM-1, ICAM-1) (114, 115). Although less studied, cecal patches and colonic patches follow similar developmental cues as described in PP development.

In contrast to PP and all other SLOs, cryptopatches start developing in early postnatal life, without the requirement of microbial signals (116). They are the most numerous lymphoid structures in the gut (30000 in humans, 1500 in mice) and homeostatically control intestinal epithelial barrier function (117). Microbial signals trigger the transition of cryptopatches to isolated lymphoid follicles (102), through the activation of CCR6^+^ ILC3 cells that respond to epithelial signals (CCL20 and IL-7) and upregulate the expression of LTαβ, triggering the differentiation of LTβR-expressing stromal cells. Stromal cell derived chemokines (CXCL13, CCL19, CCL21) and adhesion molecules (VCAM-1, ICAM-1) drive the recruitment of lymphocytes and formation of a single B cell follicle, with a germinal center and a network of follicular dendritic cells, localized under a dome epithelium. ILFs contribute significantly to T-cell independent IgA synthesis (118) and generation of regulatory T cells, that drive tolerogenic immune response towards commensalist organisms (119). Compartmentalization of SLOs in the lamina propria of the intestine and maintenance of ILC homeostasis and function is highly dependent on fibroblastic reticular cell niches (FRC) (120), primarily regulated by LTβR-signaling and CCL19. Upregulation of CCL19 and the emergence of a signature FRC population was described in inflammatory bowel diseases (121), therefore identification of FRC related druggable targets could lead to controlling sustained intestinal inflammation.

**Figure 2:**
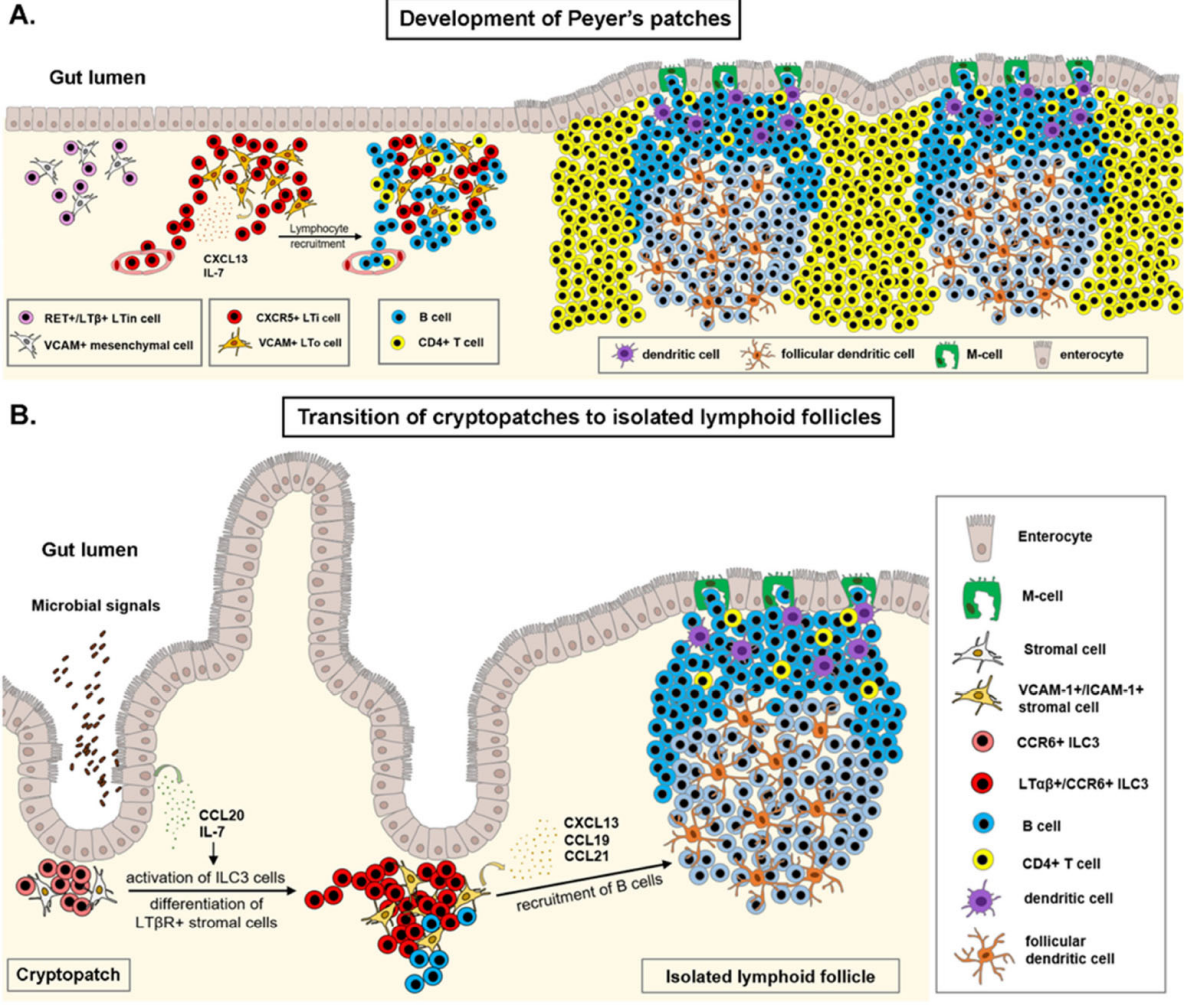
Development of gut-associated lymphoid tissue. **(A)** Establishment of the Peyer’s patch (PP) anlagen occurs during embryonic development, with the clustering of RET receptor positive, lymphotoxin-β expressing lymphoid tissue initiator (LTin) cells in the developing lamina propria of the ileum. VCAM^+^ mesenchymal cells differentiate to lymphoid tissue organizer (LTo) cells and secrete IL-7 and CXCL13 ligands. The CXCL13-CXCR5 axis drives accumulation of fetal liver derived lymphoid tissue inducer (LTi) cells to the PP anlagen, where binding of the lymphotoxin-α1β2 to its receptor, LTβR on mesenchymal LTo cells induces the expression of adhesion molecules, chemokines and cytokines. This positive feed-back loop reinforces recruitment of additional LTi cells and circulating lymphocytes. Organization of the PP anlagen results in the formation of several B-cell follicles and interfollicular T-cell zones in the lamina propria of the ileum. **(B)** Clusters of CCR6^+^ type 3 innate lymphoid cells (ILC3) at the base of intestinal crypts contribute to intestinal epithelial barrier function in early postnatal life. Upon microbial stimulus, ILC3 cells in cryptopatches upregulate the expression of lymphotoxin-α1β2, drive differentiation of VCAM-1^+^/ICAM-1^+^ stromal cells, and recruit lymphocytes to subepithelial regions, forming a single B-cell follicle at the site of infection. Illustrations were made using MS PowerPoint and Adobe Illustrator. Images were compiled by Adobe Photoshop.

Independent of the clustering of ILC3 type LTi cells, infection and chronic inflammation trigger the formation of highly structured tertiary lymphoid organs (TLOs, tertiary lymphoid structures) in the lamina propria of the GI tract. TLOs have been described to sustain inflammation and activation of auto-reactive lymphocytes and production of disease-specific autoantibodies in a number of inflammatory diseases (122). On the other hand, presence of TLOs in anti-tumor response has been reported beneficial in most solid tumor malignancies via the local presentation of tumor antigens, activation of antibody and cytotoxic responses (123). Tumor associated TLO gene expression studies offer a valuable tool in predicting therapeutic immune responses and choosing adequate anti-tumour therapies (124, 125). Induction of TLOs by therapeutic intervention in cases when the tumour microenvironment is non-permissive may facilitate lymphocyte recruitment, tumor control and better prognosis (126, 127). **Figure 2** illustrates the formation of SLOs in the GALT.

### Formation of the respiratory MALT

Mucosa associated lymphoid tissues in the respiratory tract are very heterogeneous among mammalian species. The upper respiratory tract in humans does not characteristically contain organized mucosa associated lymphoid structures. In contrast, rodents develop extensive nasal associated lymphoid tissues (NALT) upon antigenic encounter, with follicular B cell and interfollicular T cell regions (128). Similar to TLOs, inducible bronchus-associated lymphoid tissues (iBALT) are the main sites where lymphocyte priming occurs in the respiratory tract upon lung infections in humans and mice (129). They characteristically form at branch points of the bronchial tree and usually next to or surrounding a pulmonary artery. Although some mammals (pigs, goats) develop BALT structures during embryogenesis, in humans and mammals this process is triggered by respiratory infections, without the essential role of ILC3 cells. Main drivers of iBALT formation are cytokines (IL-17, IL-22) and chemokines (CCL19, CCL21) provided by surrounding epithelial, endothelial and stromal cells, γδ T cells and follicular-homing T_h_17 cells (129, 130). This results in the differentiation of follicular dendritic cells from lung fibroblasts and the recruitment of B- and T-cells in the perivascular space along the bronchi (131). Maintenance of iBALT structures in later stages relies on lymphotoxin-signaling, between LT expressing activated lymphocytes, dendritic cells and LTi cells and LTβR stromal and endothelial cells, that drive differentiation of HEVs, FDCs and new lymphatic vessels (132, 133).

**Figure 3:**
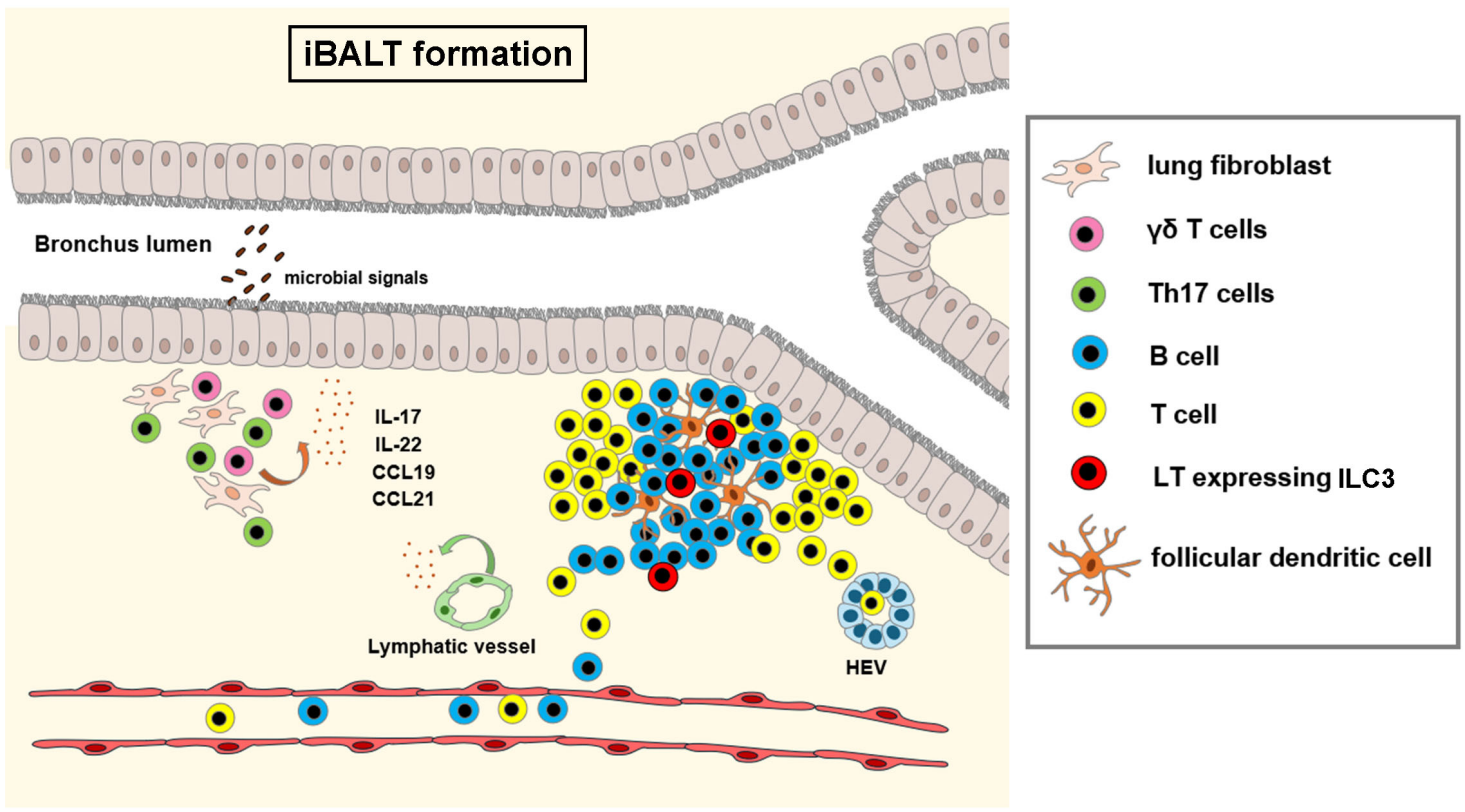
Formation of inducible bronchus associated lymphoid tissue (iBALT). Infection or chronic inflammation induce the formation of bronchus associated lymphoid tissue. Epithelial and stromal cells, γδ T cells and T_h_17 cells produce cytokines (IL-17, IL-22) and chemokines (CCL19, CCL21), that trigger the differentiation of follicular dendritic cells from lung fibroblasts and recruit circulating lymphocytes to the perivascular space at branching points of the bronchi. In later stages, ILC3 derived lymphotoxin signals drive the organization of lymphocytes, differentiation of high endothelial venules (HEV) and new lymphatic vessels. Illustrations were made using MS PowerPoint and Adobe Illustrator. Images were compiled by Adobe Photoshop.

iBALT formation is critical in adequate response to a number of pathogens (*M. tuberculosis, Pneumocystis, P. Aeruginosa, H5N1 influenza virus*) that results in short-term inflammation by early recruitment of neutrophils, conventional dendritic cells and IFNγ-mediated pathogen clearance. In contrast, chronic pathogen exposure and uncontrolled inflammation drives conversion of protective iBALT structures to pathogenic iBALT, associated with dysregulated lymphocyte proliferation, autoantibody production and chronic pulmonary inflammation induced tissue damage (allergy/asthma, chronic obstructive pulmonary disease, pulmonary arterial hypertension) (129). In non-small cell lung cancer (NSCLC), presence of NCR^+^ ILC3 has been associated with the formation of protective tumor-associated tertiary lymphoid structures (134), that are associated with favorable clinical outcome. **Figure 3** shows iBALT formation.

The level of the pharynx is an intersection point between the GI- and the respiratory systems. Humans exhibit well-developed mucosal lymphoid structures at this level. The tonsils, organized in the Waldeyer’s ring play as the first line of defense against ingested or inhaled pathogens. Covered by stratified squamous epithelium, infiltrated by a number of immune cell populations, tonsils exhibit organized follicular B-cell and interfollicular T-cell regions. Embryonic development of the tonsils follows an intrinsic developmental program, without antigen encounter; the exact molecular mechanisms however, that guide the formation of tonsil primordia are largely unknown (135). In contrast, rodents lack tonsils, but develop complex nasopharyngeal lymphoid tissue (NALT) structures along the nasal passage, with similar anatomical and functional organization to human tonsils. Covered by ciliated epithelium, NALT exhibits cellular organization into B-cell follicles under a dome epithelium and interfollicular T-cell zones. Development of the NALT is induced upon antigenic encounter after birth and relies on unique developmental pathways that do not require ILC3 type LTi cells, IL-7, CXCL13 or lymphotoxin-alpha signaling - pathways indispensable for the development of most lymphoid tissues (128, 136). Mice lacking the Id2 transcription factor, however, lack NALT structures, which demonstrates the importance of ILC cell populations, such as ILC2 cells, in the initial organization of murine NALT (137).

Mucosa associated lymphoid structures in the gut and lungs represent a dynamically rearranging, complex network of immune cells with crucial roles in physiological and pathological immune responses. In contrast to the lungs, where formation of the main mucosa associated lymphoid structure occurs upon microbial stimulus, the gastrointestinal tract harbors classical SLOs, with intrinsic developmental cues during intrauterine development. Organization and maintenance of most MALT structures are highly dependent on ILC3 type cells that drive lymphocyte recruitment and organization, orchestrate immune tolerance, and contain infections; whereas inducible MALT structures are main drivers of chronic inflammation and allergy.

## Cells of mucosal immunity and the innate immune system: a comparison

The MALT comprises a specific layer important in the immune responses, a single layer of epithelium covered by mucus and antimicrobial products, fortified by innate and adaptive components in the underlying lamina propria (138). In the GI tract, Paneth cells in the crypt produce alpha-defensins, while the epithelial cells produce beta-defensins for host protection. Intraepithelial lymphocytes reside between the epithelial cells, consisting of T cell subsets. We can discover conventional T cell subsets such as T_h_1, T_h_2, T_h_17, T_reg_, and memory T cells (139), but also B cell-enriched areas where soluble IgA is produced by plasma cells or memory B cells. In the subepithelial area, antigen-presenting cells (APCs), such as dendritic cells (DC) or macrophages, are found. Microfold (M) cells absorb antigens from the intestinal tract’s lumen and nasal mucosa, then deliver them to the dendritic cells beneath (140–142).

The mononuclear phagocyte system (MPS), comprising monocytes, macrophages, and dendritic cells, plays a significant role in innate immunity and the MALT. These cells are strategically situated in barrier tissues such as the gut, where they perform antigen sampling and immune surveillance. Within the MPS, dendritic cells are instrumental in capturing antigens and migrating to lymph nodes, where antigen presentation to T cells occurs. This process is essential for the initiation and modulation of adaptive immune responses. Moreover, macrophages contribute to the maintenance of tissue homeostasis and the resolution of inflammation through the phagocytosis of pathogens and cellular debris. In the context of the microbiome, the MPS actively participates in the crosstalk between luminal microbiota and the host influencing mucosal immunity and systemic immune responses. Mononuclear phagocytes in the MALT regulate the balance between tolerance to commensal microbes and the immune reactivity towards pathogens. By sampling bacterial antigens from the intestinal lumen, these cells can induce tolerogenic or immunogenic pathways, depending on the context and nature of microbial interactions. Due to their unrelenting drive to interact with the luminal microflora, we choose MPS cells for a more detailed introduction among cellular elements of the MALT.

### Macrophages

The process of tissue-resident macrophage development begins with the emergence of primitive macrophages in the yolk sac (143). These macrophages spread throughout embryonic tissues following the establishment of blood circulation (144). This early hematopoiesis is independent of the transcription factor Myb. However, when hematopoietic stem cells from the aorta-gonad-mesonephros colonize the fetal liver, definitive hematopoiesis begins Myb-dependent, generating all major hematopoietic lineages, including monocytes (145). These fetal liver monocytes infiltrate all peripheral tissues except for the central nervous system and develop into tissue-resident macrophages. These macrophages mostly coexist with, but can gradually outcompete, yolk sac-derived tissue macrophages. Both yolk sac-derived and fetal liver-derived macrophages are characterized by their longevity and ability to self-renew (146). In adulthood, monocytes expressing high levels of Ly6C can develop into relatively short-lived, non-self-renewing tissue-resident macrophages in organs displaying homeostatic inflammatory conditions, such as the intestine, the remodeling mammary gland, and the heart (147).

In the GI tract, there are four different subsets of macrophages: peritoneal macrophages, which support IgA production by peritoneal B1 cells; muscularis gut macrophages, which regulate smooth muscle contractions (148); intraganglionic macrophages (149, 150) and intestinal lamina propria macrophages. These intestinal lamina propria macrophages have three functions: maintaining gut homeostasis, producing cytokines that establish mucosal immunity, and uptaking antigens to present to different immune cell types (151). The only known type of macrophage in the respiratory tract is the alveolar macrophage, which phagocytizes excessive surfactants and surfactant-opsonized particles (152).

### Dendritic cells

The development of dendritic cells (DCs) is a continuous process that occurs in the bone marrow, necessitated by the need for constant renewal of mature DCs in peripheral tissues. The initial belief was that mature DCs stimulated immunity, while immature DCs promoted tolerance. Recent findings have shown that even phenotypically mature DCs can promote tolerance instead of immunity. The critical difference lies in the expression of soluble mediators such as IL-10, TGF-beta, retinoic acid, etc., and surface tolerogenic receptors like OX40L, CTLA-4, PDL-1, etc (153). In the case of conventional DC (cDC) development, hematopoietic stem cells evolve from early progenitors into more lineage-specific macrophage-DC progenitors. These further differentiate into common DC progenitors (CDPs), the first progenitors exclusive to the DC lineage. CDPs subsequently give rise to pre-DCs, which migrate from the bone marrow to peripheral organs and differentiate locally into mature cDCs.

In the GI tract, pDCs and dendritic cells within the epithelium are key players in immune surveillance. During inflammation, monocyte-derived dendritic cells (moDCs) are transient, bolstering the local immune response. Among cDC subsets, cDC1s with CD103^+^ and CD11b^-^ phenotypes, as well as cDC2s encompassing CD103^+^CD11b^+^ and CD103^-^ CD11b^+^ cells, intricately contribute to orchestrating immune balance and efficient pathogen defense in the gut. The CD103^+^CD11b^+^ cells regulate the initiation of the mucosal antibody (Ab) response to soluble flagellin (sFliC) (154) in regards to mucosal and systemic CD103^−^CD11b^+^ dendritic cells, the presence of TLR5 expression governs the sensitivity of the T cell immune response to flagellated pathogens (155). This nuanced array of dendritic cell subsets ensures a finely tuned and responsive immune system in both the respiratory and gastrointestinal tracts (156). DCs are also located in the lamina propria of the small and large intestines (these are the follicular DCs) and the gut-associated lymphoid tissue. This includes isolated lymphoid follicles, Peyer’s patches, and mesenteric lymph nodes. Dendritic cells that produce retinoic acid boost the expression of mucosal homing receptors (alpha4beta7 and CCR9) on activated T cells (157). This facilitates their subsequent journey through the lymphatic system, the bloodstream, and finally into the lamina propria of the gastrointestinal tract.

In the respiratory tract, dendritic cell populations exhibit remarkable diversity. Among them, plasmacytoid dendritic cells (pDCs) and conventional dendritic cells (cDCs) further diversify into distinct subsets, including mDC1 and mDC2 cells. Novel types of dendritic cells, including IGSF21^+^ DC, EREG^+^ DC, and TREM^+^ DC, play pivotal roles in antigen presentation and immune regulation, as indicated by Travaglini et al. (71). EREG, one of the seven cell-surface EGFR ligands, has been previously documented to protect the gastrointestinal tract from dextran sulfate sodium colitis. Furthermore, EREG expression by DC3 serves as a critical signal for maintaining fibrosis in both the skin and lungs (158). TREM-2^+^ dendritic cells maintain nitric oxide (NO) production to regulate T_h_17 differentiation negatively (159). Additionally, monocyte-derived dendritic cells (moDCs) emerge during inflammation, playing a critical role in shaping immune responses within the lung microenvironment (160), such as antigen-presenting (161). The susceptibility to infection, the induction of IL-12 production, and the capability for L. major-specific T cell stimulation by dermal monocyte-derived DCs highlight their involvement in directing the initiation of protective T helper 1 response against Leishmania (162).

The development of lung-resident plasmacytoid DCs (pDC) has yet to be addressed explicitly, but some general mechanisms of pDC development have been identified. Unlike cDCs, pDCs fully develop in the bone marrow and then migrate to peripheral organs (163, 164). Monocyte-derived DCs (moDC) have only been found in the lungs in a steady state, as these cells have long been difficult to distinguish from CD11b positive cDCs due to their similar surface markers (165). Due to their monocyte origin, moDCs rely on the same factors that monocytes depend on for their development, including the chemokine receptor CCR2 (166) and the cytokine CSF-1 (167).

### T-cells of innate immunity

In addition to conventional immune cells, mucosal tissues contain a significant number of innate-like cells and unconventional T cells. These innate-like cells help maintain the barrier function by detecting changes in the tissue environment and releasing effector cytokines. Unlike conventional T cells, Innate Lymphoid Cells (ILCs) do not rely on antigen-specific T-cell receptors and Recombination-Activating Genes (RAG) for their development. Instead, they depend on cytokine signaling through the common gamma chain encoded by the interleukin-2 receptor gamma (105). These cells are categorized into three main groups (ILC1, ILC2, and ILC3) based on their cytokine profiles and transcription factor dependencies. ILC1s primarily produce IFN-γ, ILC2s secrete IL-5 and IL-13, and ILC3s release IL-17 and IL-22 (168). These cytokines are pivotal for maintaining mucosal homeostasis, responding to microbial challenges, and modulating tissue repair and inflammation (168). In the gut, ILCs contribute to the integrity of the epithelial barrier and orchestrate immune responses against pathogenic microbes while maintaining tolerance to commensals and dietary antigens (169, 170). ILC3s, in particular, interact with the gut microbiota and epithelial cells to promote mucosal healing through the secretion of IL-22 (171, 172). Predominantly found in the lungs, ILC2s produce cytokines such as IL-5 and IL-13, which are crucial for promoting airway eosinophilia, mucus production, and smooth muscle contraction protective against helminths, but also contributing to the pathogenesis of asthma and allergic inflammation, licensing DCs to potentiate T_h_2-responses (173–176). Furthermore, ILC2s also secrete IL-9 and amphiregulin, associated with tissue-protection after influenza virus infection (177). ILC1s and ILC3s, though less abundant, are involved in defense against bacterial and viral infections, producing IFN-γ, IL-17 and IL-22 (178–182).

Gamma-delta (γδ) T-cells in the gut and the MALT are specialized subsets of T-cells characterized by their distinct T-cell receptor (TCR) which differs from the alpha-beta (αβ) TCR found on conventional T-cells (183, 184). Predominantly located in the epithelial layer of the GI tract, γδ T-cells function as a first line of immune defense, crucial for maintaining epithelial integrity, facilitating wound healing, and providing rapid responses to pathogenic invasion, capable of modulating the gut microbiome (185–187). In the gut, γδ T-cells are involved in the surveillance against malignantly transformed cells and the control of infections through the secretion of cytokines such as IFN-γ and IL-17, aiding localized inflammatory responses and modulating the activity of other immune cells, including macrophages and neutrophils (188). In the lungs, by producing growth factors, γδ T-cells support the regeneration of lung tissue following injury and contribute to epithelial repair and maintenance of barrier integrity (189). In diseases like COPD and asthma, γδ T-cells can have dual roles: they may limit infection and promote repair, but their dysregulation can lead to chronic inflammation and tissue damage, exacerbating disease pathology (190, 191). Furthermore, Latest research found that Intratumoral γδ T-cells possessed stem-like features and elicited cytolysis and T_h_1 cellular function improving survival in lung cancer (192).

### Crosstalk between the MALT of the gut and the lungs

The immune communication between the gut and lung is a bidirectional process. For example, inoculating the nose with *Salmonella* triggers a *Salmonella*-specific immune response in the gut, which relies on lung dendritic cells. This implies that dendritic cells and macrophages in the GI and upper respiratory tracts can move or even transfer information from one immunization location to another. For example, lung dendritic cells induce the migration of protective T cells to the GI tract (193). Germ-free (GF) animals experience a slower elimination of a harmless bacterium following a systemic challenge. The extent of the myeloid cell population in the bone marrow is closely linked to the diversity of the gut microbiota.

The gut microbiota influences hematopoiesis by modulating several aspects of the bone marrow microenvironment, therefore indirectly influencing disease progression and pathology. In response to NOD1 ligands from the microbial community, bone marrow stromal cells produce hematopoietic cytokines (IL-7, Flt3L, SCF, TPO, and IL-6) (194). Similarly, CX3CR1^+^ monocytes residing in the perivascular area of the bone marrow sense bacterial DNA through the systemic circulation and secrete TNF-α, IL-1β, and IL-6 (195), that leads to the expansion of the hematopoietic stem cell pool. HSC expansion is also directly influenced by microbiota-derived metabolites found in circulating blood. Lactate activates SCF expression of LepR^+^ cells in sinusoidal blood vessels and induces the proliferation of HSCs (196). Similarly, bacterial-derived short-chain fatty acids (SCFAs) lead to the generation and activation of highly phagocytic macrophage and dendritic cell precursors that seed to the lungs and protect against allergic inflammation (197). In contrast, alteration of commensal bacterial populations via oral antibiotic treatment triggers elevated serum IgE concentrations, increased numbers of circulating basophil granulocytes and allergic inflammation (198). Alterations in the microbiota due to dysbiosis, obesity, or antibiotic use could disrupt the communication between hematopoiesis and the microbiota, potentially worsening inflammatory or infectious conditions in the host (199).

ILCs also play a significant role in the gut-lung axis. Evidence suggests that ILCs, especially ILC2s and ILC3s, can migrate between these sites reciprocally and modulate immune responses in respiratory diseases controlled by the gut microbiome (200, 201). For example, gut-derived ILCs can influence pulmonary inflammation and pathologies mediated via systemic circulation and possibly through microbial metabolites and cytokines across distant mucosal sites (202). γδ T-cells might also be implicated in mediating systemic immune responses that link GI health to pulmonary health by microbial metabolites or translocating microbes that can activate these cells (187, 203). **Table 1** summarizes the phenotypic distribution of macrophages and DCs in the MALT of the gut and the lungs.

**Table 1:** The MPS system of the gut and the lungs.

System	Name	Type	Function	References
Dendritic cells				
Lung	plasmacytoid dendritic cells	pDC	antigen presenting	(71)
	conventional dendritic cells	cDC - mDC1	release several proinflammatory cytokines	(204)
	conventional dendritic cells	cDC - mDC2	release several proinflammatory cytokines	(204)
	IGSF^+^ dendritic cell	DCIGSF21^+^	unknown	(71)
	EREG^+^ dendritic cell	DCEREG^+^	maintaining fibrosis	(158)
	TREM^+^ dendritic cell	DCTREM^+^	downregulation of T_h_17 differentiation	(159)
	monocyte-derived dendritic cells	moDC	T_h_1 activation with IL-12 production	(162)
	monocyte-derived dendritic cells	moDC	antigen presenting	(161)
Gut	follicular dendritic cell	fDC	RA production → CCR9 upregulation on T cells	(157)
	plasmacytoid dendritic cell	pDC	antigen presenting	(154)
	monocyte-derived dendritic cells	moDC	antigen presenting	(154)
	monocyte-derived dendritic cells	moDC	CCR2 and CSF1 dependent T cell homing	(154)
	CD103^+^/CD11b^-^ conventional dendritic cell	cDC1CD103^+^/CD11b^-^	sensing pathogens and tissue damage and activation of naive CD8^+^ T cells	(205)
	CD103^+^/CD11b^+^ conventional dendritic cell	cDC2CD103^+^/CD11b^+^	mucosal antibody response to soluble flagellin	(154)
	CD103^-^/CD11b^+^ conventional dendritic cell	cDC2CD103^-^/CD11b^+^	T cell recruitment to flagellated pathogens	(155)
Macrophages				
Lung	alveolar macrophage	aMø	surfactant phagocytosis	(152)
Gut	peritoneal macrophage	pMø	IgA production by B1 cells	(148)
	muscularis gut macrophages	mgMø	smooth muscle contraction regulation	(148)
	intraganglionic macrophages	igMø	enteric neuroinflammation	(149, 150)
	intestinal lamina propria macrophage	ilpMø	homeostasis cytokines production antigen presenting	(151)

The reciprocal exchange of signals between the gut and lungs underscores the interdependency of mucosal immunity, wherein immune cells relay information and reactions across distant anatomical sites. This dynamic interplay is shaped by factors like the gut microbiota, which can shape hematopoiesis and influence immune cell dynamics in the bone marrow, thereby impacting overall immune function. A comprehensive understanding of these interactions and pathways within the MALT is crucial for targeted interventions to modulate immune responses and effectively combat inflammatory or infectious diseases. Continued research into the development, functionality, and regulation of immune cells within MALT promises to yield valuable insights into therapeutic approaches for various mucosal-related disorders.

## The gut and the lung microbiome and its implication in the gut-lung axis

The human microbiome, constituting trillions of commensal, mutualistic, and pathogenic s microorganisms, plays an indispensable role in the health and disease of its host. Once distinct and isolated kingdoms, the gut and lung microbiomes are now recognized as intimately linked ecosystems engaged in a continuous crosstalk. The gut microbiome is crucial for metabolic functions, synthesis of vitamins, and development of the immune system (206–208). In contrast, the lung microbiome, once considered sterile, has been revealed to host a less diverse but dynamic community influenced significantly by the inhalation of environmental air (209, 210). Unlike the gut’s relatively stable environment, the lung exhibits a more inhospitable landscape for microbial colonization due to its high oxygen levels, constant immune surveillance, and mucociliary clearance (211). Yet, both microbiomes share functional similarities in their influence on the host’s immune system, albeit through different mechanisms and with varying impacts on host physiology. Recent studies highlight this microbial gut-lung axis as an emerging phenomen, suggesting a bidirectional communication where dysbiosis in one site can influence disease processes in the other (1, 4, 212). One particular study even directly identified the role of the intestinal microbiota in safeguarding against pneumococcal pneumonia by boosting the function of alveolar macrophages (213).

### Sampling and analysis

The analysis of these microbiomes employs a range of techniques from culture-based methods to advanced metagenomics, transcriptomics, and metabolomics. High-throughput sequencing technologies include 16S rRNA gene sequencing for bacterial identification, whole-genome shotgun sequencing (Metagenomics) providing insights into the composition and functional capabilities of these microbial communities (214–216), and long-read sequencing (LRS) as an innovative method, whose strongest advantage lies in its enhanced accuracy in characterizing genomic landscapes, particularly in areas such as structural variations, repeat expansions, and complex genomic regions (216, 217). However, the complexity of sampling, particularly from the lung where invasive procedures are often required, poses challenges to accurately characterizing its microbiome (218–222). Sequencing of sputum and bronchoalveolar fluid samples often yield disparate results and contaminating microbiota can alter analyses (223–225). The concept of the gut-lung axis from a microbiome perspective (226) introduces a paradigm where gut microbiota alterations mediated by immune modulation, microbial metabolites, and molecular mimicry can affect lung health (197, 227) and vice versa, implicating the existence of a presumptive lung-gut axis, though the latter lacks conclusive scientific evidence so far (19, 228, 229). Microaspirations stand out as a primary conduit for the translocation of oral microbes to the lower respiratory tract, contributing to the lung microbiome’s composition and potentially influencing respiratory health. Inhalation of small droplets containing oral microbiota into the lungs can alter the pulmonary microbial landscape, impacting the development and progression of chronic respiratory conditions such as chronic obstructive pulmonary disease (COPD) and asthma (230–232). This phenomenon underscores the critical role of the oral-lung axis in respiratory health.

### Taxonomy and diversity

The gut reigns supreme in terms of microbial abundance and diversity, where *Bacteroidetes* and *Firmicutes* dominate alongside with a minor representation of *Actinobacteria, Proteobacteria*, and *Verrucomicrobia. Bifidobacteria* and *Lactobacilli* (from the phyla *Actinobacteria* and *Firmicutes*, respectively) play key roles in nutrient digestion and immune modulation (233). *Prevotella* and *Faecalibacterium* genera are essential for processing dietary fibers and producing short-chain fatty acids (SCFAs) (234). In contrast, the lung presents a sparser landscape, with only around 100 bacterial species colonizing the lower airways (235), where *Firmicutes* and *Proteobacteria* are the primary residents, with their composition heavily influenced by environmental factors like inhaled particles and the oral microbiome (206). The lung microbiome includes genera such as *Streptococcus*, *Haemophilus*, *Veillonella* and *Pseudomonas*, adapted to the moist, oxygen-rich environment of the respiratory tract. Unlike the gut, where a dense microbial population is beneficial, the lungs require a balance to prevent infections and maintain efficient gas exchange (236). The gut microbiome is renowned for its high alpha diversity and richness. Alpha diversity refers to the variety and abundance of species within a specific ecosystem. In the context of the microbiome, it environs the range of microbial genera present in a particular body site and their relative abundance. Richness, a component of alpha diversity, simply counts the number of different species present, regardless of abundance. High alpha diversity and richness, including predatory species in the gut (237) is generally associated with good health (238), correlating with resilience to pathogenic colonization (239), and a balanced immune response, including anti-cancer immunity (25, 26). Concerning the lung microbiome, an overall low-diversity system, the impact of alpha-diversity is still under debate and may not correlate directly with pulmonary health (211, 240) and its significant change might only be associated with severe pathologies and iatrogenic factors (241, 242).

### Variability

Regarding its intraindividual variation, the gut microbiome is relatively stable in case of normalized diets and lack of disease (243–245), however, abundance of functional pathways may show a greater variability (246). In the case of the lung microbiome, due to sampling limitations evidence is scarce, and mostly been studied in conjunction with environmental factors and lung diseases implicating a greater intraindividual heterogeneity (211, 247, 248). Interindividual variation of the gut microbiome mainly depends on human diet (249–252) strongly affected by geography (253, 254), race and culture (255, 256) and can be also reproduced in experimental mice (257). Lately it has been shown that it is a considerable factor in host susceptibility to pathogens (258). Lung microbiome heterogeneity across people has mainly been studied in disease and has been linked to the oral microbiome (211, 259). Exposure to smoke is a well-known detrimental factor in lung health and has been associated with altered lung microbiota by a large piece of scientific literature (260–262). Interestingly, smoking affects gut commensals too, raising intrigue concerning its role in the gut-lung axis (263, 264).

### Functionality

Despite their compositional differences, both microbiomes share fundamental functionalities. They contribute to host metabolism by extracting energy from dietary components and synthesizing essential nutrients (265, 266). They also play a crucial role in immune regulation, shaping both innate and adaptive responses (226, 267). Gut microbes produce SCFAs that signal immune cells in the gut and remotely in the lungs, influencing inflammatory responses and allergic reactions (268). Disruptions in either microbiome can lead to inflammatory bowel disease (IBD), asthma, and other chronic inflammatory conditions (269, 270). Microbial metabolites like trimethylamine N-oxide (TMAO), generated by gut bacteria, can travel to the lungs and impact cardiovascular health (271) or the blood-brain barrier (272, 273). Moreover, studies suggest that the gut microbiome can influence susceptibility to respiratory infections like influenza and pneumonia (274, 275). Animal models further elucidate pathways of communication: germ-free mice, lacking a gut microbiome, exhibit increased susceptibility to lung infections (276), Conversely, manipulating the gut microbiome with probiotics or antibiotics can impact lung inflammation and allergic responses (277, 278). The interconnectedness of the gut and lung microbiomes makes a somewhat asymmetrical bidirectional communication possible underpinning the concept of gut-lung axis. The balance between these microbiomes is crucial for health, suggesting that targeted modulation could offer new therapeutic approaches for treating chronic diseases and improving overall well-being. Comparison of the gut and the lung microbiomes is shown in **Table 2**.

**Table 2:** Comparison of the Gut- and the Lung Microbiomes.

Characteristic	Gut Microbiome	Lung Microbiome
Composition	Dominated by bacteria from the phyla *Firmicutes* and *Bacteroidetes*.	Primarily composed of members from the phyla *Firmicutes, Bacteroidetes*, and *Proteobacteria*.
Predominant Genera	*Bacteroides, Lactobacillus, Prevotella, Faecalibacterium, Clostridium*, and *Bifidobacterium*	*Streptococcus, Prevotella, Veillonella*, and *Pseudomonas*.
Response to External Factors	Diet, antibiotics, and lifestyle can significantly alter composition and function.	Influenced by air quality, smoking, and exposure to pathogens.
Host Interaction	Extensive interaction with the immune system, influencing systemic immunity.	Direct interaction with the respiratory immune system, affecting local and partly systemic immunity.
Diversity	High	Lower than gut
Abundance	Extremely high, with bacterial cells outnumbering human cells approximately 10:1 in the colon.	Lower than the gut, with microbial load increasing from the upper to lower respiratory tract.
Richness	Very rich, harboring hundreds to thousands of species.	Less rich compared to the gut, with dozens to hundreds of species detected.
Intraindividual Variability	Relatively stable but can be influenced by diet, antibiotics, and disease.	More variable than gut, influenced by environmental exposure and lung health status.
Interindividual Variability	High, influenced by genetics, age, diet, lifestyle, and environmental factors.	Also high, but less studied. Influenced by age, smoking status, environmental exposures, and health status.
Geographic Heterogeneity	Exhibits significant variation based on dietary habits, lifestyle, and environmental factors of different populations.	Less is known, but preliminary studies suggest variation rather based on environmental exposure and lifestyle factors.
Functional Role	Critical in digestion, immune modulation, and protection against pathogens. Involved in synthesis of vitamins and metabolism of dietary compounds.	Important in immune response and maintaining respiratory health. Involved in protecting against pathogens and modulating inflammation.
Response to Treatment	Dramatically affected by antibiotics, which may lead to dysbiosis. Responsive to probiotics and dietary interventions.	Antibiotics can alter lung microbiome composition, potentially affecting respiratory health. The impact of probiotics is less clear and under investigation.
Research Challenges and Gaps	Complexity of interactions between diet, microbiome, and host health. Difficulty in distinguishing cause from correlation in disease.	Sampling challenges due to lower biomass and contamination from upper respiratory tract. Limited understanding of the lung microbiome’s role in health and disease.
Microbial-host Interactions	Plays a significant role in shaping the immune system and metabolic health. Influences host gene expression.	Critical for immune tolerance and defense against pathogens. May influence lung disease progression and response to therapy.
Emerging Therapeutic Approaches	Fecal microbiota transplants (FMT), microbiome-targeted therapies (phage-therapies), and engineered probiotics.	Phage therapy, microbiome modulation through diet or inhaled probiotics, and targeted antimicrobial therapies.

## Gut permeability: a pivotal factor in systemic immunity and in lung disease

Intestinal permeability and the intestinal barrier are closely related concepts that together play a crucial role in maintaining the health of the GI system. The intestinal barrier is a sophisticated multi-layered system comprising an outer “physical” barrier and an internal “functional” immunological barrier. The interplay between these two barriers ensures the maintenance of balanced permeability (279). Intestinal permeability is the regulation of substances moving from the GI tract into the body. The intestines naturally possess a level of permeability, facilitating the passage of nutrients while simultaneously serving as a barrier to prevent potentially harmful substances, such as antigens, from exiting the intestine and spreading throughout the body (280).

### Permeability in health and disease

Luminal products can pass the intestinal epithelium in several ways. The pathways depends on factors like the size, hydrophobicity and other physico-chemical properties. There are four pathways for substances to cross the intestinal lining: (a) the transcellular route is taken by small hydrophilic and lipophilic compounds; (b) the paracellular route is for ions, water, and larger hydrophilic compounds (400–600 Da up to 10–20 kDa) regulated by tight junction proteins; (c) transcellular active transport is for sugars, amino acids, and vitamins, requiring specific transporters and energy; (d) endocytosis and basolateral exocytosis are for larger peptides, proteins, large bacterial components, or even whole bacteria (281, 282). Under physiological conditions, an intact intestinal barrier serves as a safeguard against the transmission of pathogens, pro-inflammatory substances, and antigens into the internal environment. Conversely, compromised intestinal integrity facilitates their entry, potentially triggering disease or inflammation (283). In reality, intestinal permeability is a barrier associated with both the commensal microbiota in the intestine and components of the mucosal immune system. Various factors have the potential to modify intestinal permeability, including changes in the gut microbiota, disruptions in the mucus layer, and damage to the epithelium. These alterations may lead to the movement of luminal contents into the deeper layers of the intestinal wall. Furthermore, lifestyle and dietary factors, such as the consumption of alcohol and energy-dense Western-style diets, have been identified as contributors to increased intestinal permeability (284–286). There are several ways to measure intestinal permeability. One common approach involves using markers or tracers to assess the passage of substances through the intestinal barrier. Some techniques include: (a) lactulose/mannitol test which measures the absorption of these sugars. Increased levels of lactulose in urine indicate higher permeability; (b) fluorescein-isothiocyanate-labeled dextran (FITC-dextran) which is a non-invasive, affordable technique for quantifying and monitoring intestinal permeability in real time; (c) PEG (polyethylene glycol) test involves the administration of PEG with different-sized molecules, and assessing their presence in urine provides insights into permeability; (d) Ussing-chamber technique utilizes an Ussing chamber to measure the passage of molecules across isolated segments of the intestine; (e) Transepithelial/transendothelial electrical resistance (TEER) is a highly sensitive and accurate method for determining the integrity and permeability of a cellular monolayer, can be used to monitor living cells at various stages of development and differentiation. (f) confocal Laser Endomicroscopy (CLE) is a more advanced method using microscopic imaging to visualize real-time changes in the intestinal barrier (287–290).

### Permeability and the gut microbiome

There is a strong correlation between increased permeability of the intestinal epithelial barrier and the gut microbiome. Researchers studied transgenic mice with intestinal epithelial-specific constitutively-active myosin light chain kinase (CAMLCK) expression. Increasing intestinal permeability was observed due to the MLCK-dependent regulation of tight junctions. Analysing the wild-type (WT) and CAMLCKTg pups microbiome, they observed a distinction in microbiomes based on the pup’s genotype rather than the dam’s. The microbiomes of CAMLCKTg mice showed an increase in *Clostridium* and a decrease in *Bacteroidetes, Enterococcus spp*, and *Prevotella*. Thus elevated intestinal permeability has the potential to induce dysbiosis-like-microbiome shifts (291). Therefore, the relationship between the two factors works in both directions: an elevation in permeability encourages dysbacteriosis, and alterations in the microbiota can likewise influence intestinal permeability (292–294). The correlation between the compromise of the intestinal barrier and the disturbance of the gut microbiome is being increasingly acknowledged as significant contributors to various pathophysiological conditions. These conditions include irritable bowel syndrome (IBS) with a lower fecal *Lactobacillus*, higher *Escherichia coli, Bifidobacterium* and *Enterobacter* compared to the healthy controls (295). In IBD the microbiota is identified by an increase in *Bacteroidetes* and *Proteobacteria*, along with a decrease in *Firmicutes* compared to those without the condition. Notably, the levels of *Faecalibacterium prausnitzii*, a highly metabolically active commensal bacterium, are diminished in individuals with IBD (296, 297). Chronic liver diseases are also associated with worsening of dysbiosis. There is a noticeable decline in bacterial diversity and an increase in the relative abundances of *Enterobacteriaceae* and *Enterococcaceae*. These groups are more susceptible to gut translocation and are considered potentially pathogenic (298). In a mouse model with leptin deficiency (ob/ob), researchers observed a decreased abundance of the *Bacteroidetes* phylum and a notable increase in *Firmicutes* levels, thus obesity is also associated with gut dysbiosis (299, 300). Diabetes mellitus has also showed an increased *Clostridium hathewayi*, *Clostridium symbiosum* and *Escherichia coli* levels (301). Neuropsychiatric disorders with a depletion of *Lactobacillus* spp. can results in T helper cell-mediated immune dysregulation and cognitive dysfunction (302–304).

### Permeability and the gut-lung axis

While the gut and lungs have distinct anatomies, the presence of potential anatomical communications and pathways associated with their respective microbiota has strengthened the concept of a gut–lung axis (4). It exerts its influence on both the gut and lung immune systems through local or long-reaching interactions, engaging in pathways involving CD8^+^ T cells (305), T_h_17 cells (306, 307), IL-25 (308), IL-13 (309), prostaglandin E2 (310), and NF-κB (311). Simultaneously, the lung microbiota plays a role in mucosal immunity and contributes to immune tolerance by recruiting neutrophils, inducing the production of pro-inflammatory cytokines via Toll-like Receptor 2 (TLR2), and releasing antimicrobial peptides like β-defensin 2, stimulated by T helper 17 (T_h_17) cells. Additionally, the lung microbiota also has an impact on the gut immune system, for example influenza infection leads to an elevated proportion of *Enterobacteriaceae* and reduced abundances of *Lactobacilli* and *Lactococci* in the gut (312). Similarly, the instillation of lipopolysaccharide (LPS) in the lungs of mice is linked to disturbances in the gut microbiota (4, 313).

Several studies have revealed a close relationship between the gut microbiota and pulmonary disorders. Studies indicate that both the composition and functionality of the gut microbiota undergo significant alterations in individuals with lung conditions, including pneumonia (314), lung cancer (315), asthma (316), and tuberculosis (317), when compared to those in healthy individuals. Several studies showed that gut dysbiosis play a role in the initiation and the progression of lung cancer. The mechanisms through which this occurs involve genotoxicity, systemic inflammation, and impaired immune surveillance (318) and is elaborated in the next chapter. Dysbiosis in the gut has the potential to compromise the function of the intestinal mucosal barrier, elevating the permeability of the intestinal mucosa (319, 320).

**Figure 4:**
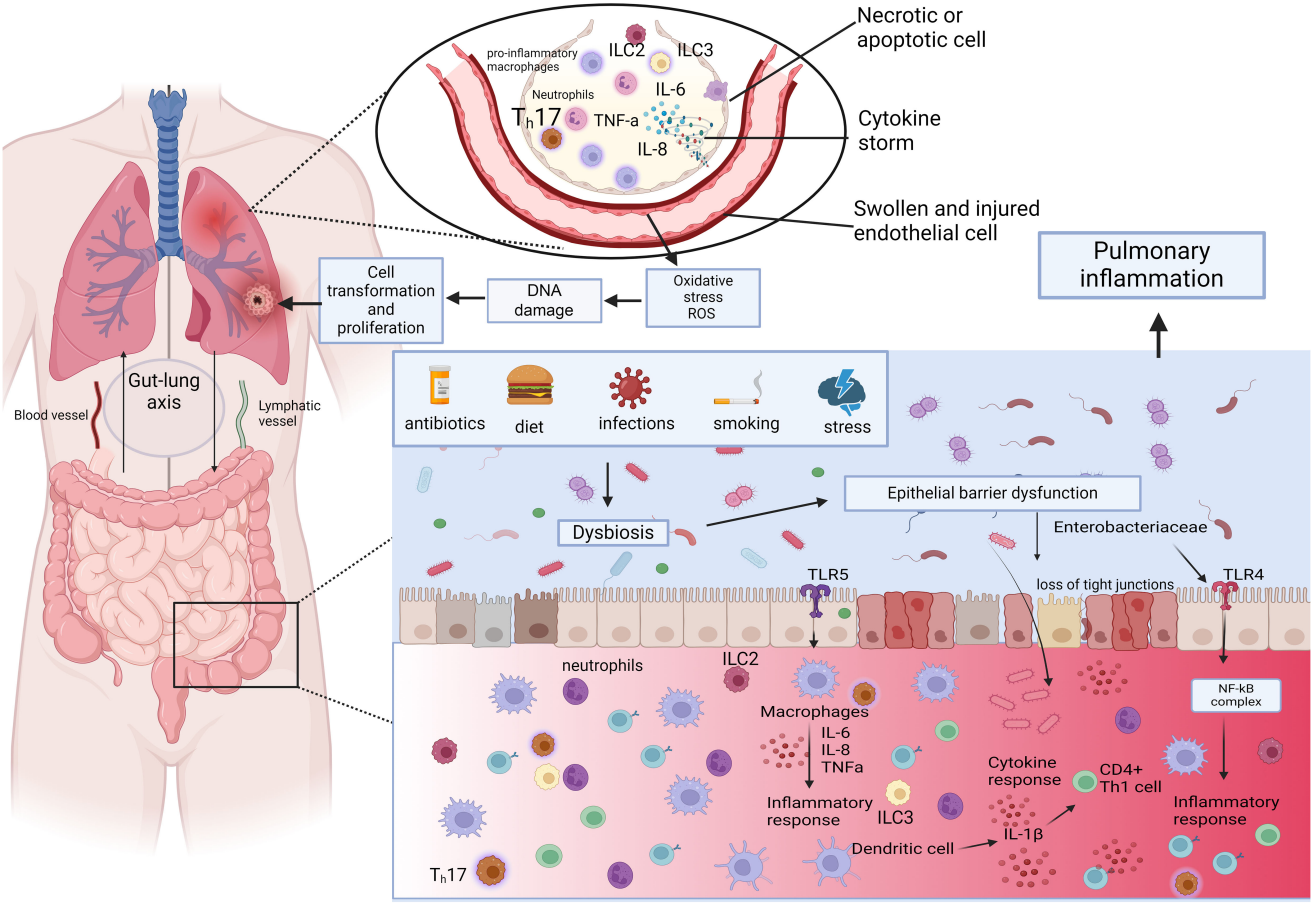
The role of gut dysbiosis through the Gut-lung axis. Dysbiosis impairs epithelial barrier function and elicits proinflammatory response. Gut dysbiosis marked by a notable rise in *Enterobacteriaceae* activates TLR4 in the intestine, which elevates IL-1β levels in the peripheral circulation, transmits inflammatory signals to the lungs, and activates the NF-κB pathway. This process triggers oxidative stress and inflammation, contributing to lung pathology through the regulation of the intestinal barrier. ILC2s, ILC3s, and T_h_17 cells that migrate from the gut to the lungs have been shown to also impact respiratory immunity. Illustrations were made using MS PowerPoint and Adobe Illustrator. Images were compiled by Adobe Photoshop.

Increased intestinal permeability can be attributed to invading microorganisms and their metabolites, initiating inflammation at both local and systemic levels. Hence, our hypothesis posits that disruptions in intestinal microbes and their metabolites could cause chronic systemic inflammation, consequently contributing to the onset and progression of lung cancer (321, 322). Microorganisms and their byproducts entering the intestinal mucosa trigger TLRs, generating inflammatory mediators. These components participate in pulmonary inflammation through the lymphatic and blood circulation pathways. Researchers showed that gut dysbiosis marked by a notable rise in *Enterobacteriaceae* activating TLR4 in the intestine, inducing inflammation. This process elevates IL-1β levels in the peripheral circulation, transmits inflammatory signals to the lungs, and activates the NF-κB pathway, ultimately leading to pulmonary inflammation (323), In a similar way, researchers observed that dysbiosis in the intestinal microbiota has the capacity to influence the TLR4/NF-kB signaling pathway. *Enterohemorrhagic E. coli* has the ability to either activate or suppress NF-κB through the Type III Secretion System (T3SS), potentially involving TLR activation (324). This, in turn, triggers oxidative stress and inflammation, contributing to lung pathology through the regulation of the intestinal barrier (325). Additionally, lung cancer patients show reduced levels of *Kluyvera, Escherichia-Shigella, Dialister, Faecalibacterium*, and *Enterobacter*, while *Veillonella, Fusobacterium*, and *Bacteroides* are significantly elevated compared to healthy individuals (326). Dysbiosis is also associated with increased zonulin release, for example *Escherichia coli, Prevotella, Pseudomonas*, and *Salmonella* spp., induce intestinal zonulin release, whereas others, mostly Gram-positive strains, such as *Bifidobacterium* and *Lactobacillus* spp., decrease zonulin levels (327). Zonulin has the ability to increase mucosal permeability by reversibly affecting the structure of tight junctions (328). Under physiological conditions, zonulin is involved not only in the small intestine but also across various extraintestinal epithelia (329). Researchers observed that zonulin could potentially play a role in pathological conditions characterized by disrupted intercellular communication, such as malignant transformation and metastasis (330). **Figure 4** demonstrates key mechanisms depending on gut permeability through the gut-lung axis.

In conclusion, changes in the gut microbiota and the disruption of the intestinal barrier have the potential to modify intestinal permeability. Increased intestinal permeability can lead to the dissemination of microbial products and their metabolites from the gut into the bloodstream, known as microbial translocation a common cause for systemic chronic inflammation (331, 332). Chronic inflammation is a well-known risk factor for cancer development, including lung cancer. Thus, the gut microbiome’s impact on systemic inflammation not only affects pulmonary diseases but also plays a crucial role in the oncogenesis and progression of lung cancer. Understanding how the microbiota co-evolves with tumors during cancer development and progression to identify the changes in bacterial composition, quantity, diversity and metabolic activity can be used as reliable biomarkers for lung cancer diagnosis. This connection underscores the relevance of the gut-lung axis in understanding pulmonary malignancies, whose development equally depends on the local inflammatory microenvironment and histological-immunological niche, as on genetic and environmental drivers.

## The role of the gut microbiome in the development of lung cancer

The discussion of the gut-lung axis strongly warrants the inclusion of lung cancer development due to its significant epidemiological impact as one of the leading causes of cancer-related deaths worldwide (333, 334). The gut microbiome, through its modulation of systemic inflammation and immune responses (335), plays a critical role in this context, mostly mediated by chronic inflammation, and driven by gut dysbiosis with increased permeability (331, 335). All these factors foster a pro-tumorigenic environment in distant organs, including the lungs (336, 337). Furthermore the enormous load of circulating metabolites and SFCAs produced by gut microbes may have a profound role in the establishment of the tumor- and premetastatic niche by the modulation of the local immune microenvironment (338–340). Emerging evidence concerning the gut microbiome’s role in lung cancer dynamics opens new avenues for targeted therapeutic strategies, aiming to manipulate the gut microbiota to counteract systemic inflammation, bolster anti-tumor immunity and prevent lung cancer development.

### Preclinical studies

As of today, the effects of various bacterial species on murine lung cancer development have been primarily investigated in relation to the combination of gut microbiome with chemotherapeutic or immunotherapeutic treatments, or with oral administration of antibiotics. Daillére et al. noted that combining *Lactobacillus johnsonii* and *Enterococcus (E.) hirae* with chemo- and antibiotic treatments in MCA205 tumor cell-injected mice led to reduced tumor sizes compared to controls (341). However, in the absence of chemotherapy, *E.hirae* did not impact natural tumor growth. Similarly, mice receiving *Lactobacillus* and *Akkermansia muciniphila* with cisplatin had smaller lung tumors compared to other treatments (342, 343). Separate studies have investigated the role of commensal gut flora in lung cancer-associated cachexia in mice, a condition that significantly raises mortality rates in lung cancer groups (344, 345).

Hagihara et al. identified specific commensal bacterial species without the administration of any treatment, which included a decreased relative abundance of *Enterorhabdus* spp., *Parvibacter* spp.*, Muribaculaceae, Desulfovibrionaceae, Ligilactobacillus* spp., and *Eubacterium brachy* in C57BL/6J mice compared to controls (346). In contrast, *Gastranaerophilales* spp.*, Lachnospiraceae, Monoglobus*, spp., and *Ruminococcaceae* were significantly more abundant in the lung cancer group. Feng et al. reported a decrease in the *Acutalibacter* genus early in lung cancer progression, decrease in *Lachnospiraceae* at later stages, and identified biomarkers like *Akkermansia muciniphila* in Lewis lung cancer (LLC) mice (347). *Lactobacillus taiwanensis, Clostridium, Desulfovibrio*, and *Eggerthellaceae* were more abundant in the lung cancer group. Zhu et al. discovered variations in gut microbiome composition at different stages of tumorigenesis, with certain bacterial families being more prevalent in groups with slower tumor growth (348). An enrichment of *Akkermansia, Bifidobacterium, Verrucomicrobiales*, and *Actinobacteria* in the slow tumorigenesis groups was observed, while *Bacteroidaceae, Flavobacteriaceae, Helicobacteraceae*, and *Enterobacterales* were decreased according to the LLC mouse study.

### Clinical studies

When assessing human case-control studies, prominent research suggests the role of *Akkermansia muciniphila* in contributing to the malignant progression of lung cancer, which is contradictory with the results of the preclinical study of Zhu et al. (315, 348). While Zheng et al. found it to be enriched in the advanced stage non-small cell lung cancer (NSCLC) group compared to healthy patients, Zhu et al. mentioned that *Akkermansia* has the potential to inhibit lung cancer through direct interaction with the tumor. At the phylum level, the results regarding the role of *Verrucomicrobia* bacteria in lung cancer development are also controversial, with studies by Liu et al., and Zhang et al. not yielding relevant results (326, 349). However, *Firmicutes* and *Actinobacteria* have been shown to play an obviously crucial role in reducing tumor size in NSCLC development (315, 326, 349, 350). On the other hand, *Bacteroidetes, Cyanobacteria, Fusobacteria, Lentisphaerae, Proteobacteria*, and *Spirochaetes* phyla showed an overall increased abundance in gut samples of lung cancer patients compared to healthy controls (315, 326, 349). Liu et al. described *Bacteroidetes* phyla as being significantly decreased in small-cell lung cancer (SCLC) patients, and Zhang et al. examined a significantly reduced abundance of *Proteobacteria* in NSCLC. Although, taking all publications into account, these phyla remain as tumor-potentiating biomarkers.

Further analysis at the genus level provides a more diverse picture of potential gut bacterial species in lung cancer development, according to recent human case-control studies. *Bacillus, Bacteroides, Clostridium, Coriobacteriaceae, Fusobacterium, Megasphaera, Oscillospira, Prevotella*, and *Synergistes* genera were overall significantly enriched in lung cancer patients (315, 326, 349, 351, 352). Moreover, *Lactobacillus, Veillonella*, and especially *Enterococcus* showed significantly increased abundance in the lung cancer group (350, 352, 353). On the contrary, *Bifidobacterium, Dialister, Faecalibacterium, Roseburia*, or for instance *Ruminococcus* were all decreased in the lung cancer patients compared to healthy participants (315, 326, 350, 354, 355). This depletion of the beforementioned genera is parallel to the current findings in the field, mentioning *Bifidobacterium, Faecalibacterium*, and *Ruminococcus* being beneficial genera in the gut, and showing decreased abundance in lung cancer patients’ stool samples (17, 349). Botticelli et al. mentioned the role of *Streptococcus* spp., showing increased abundance in lung cancer, however, Zheng et al. described it as being underrepresented in the lung cancer group, creating controversy (315, 351). Comparing the two results, in the study by Botticelli et al., a significant limiting factor was the small sample size, whereas Zheng et al. mentioned the smoking history of patients as a potential confounding factor and smoking itself can increase the risk of lung cancer.

**Figure 5:**
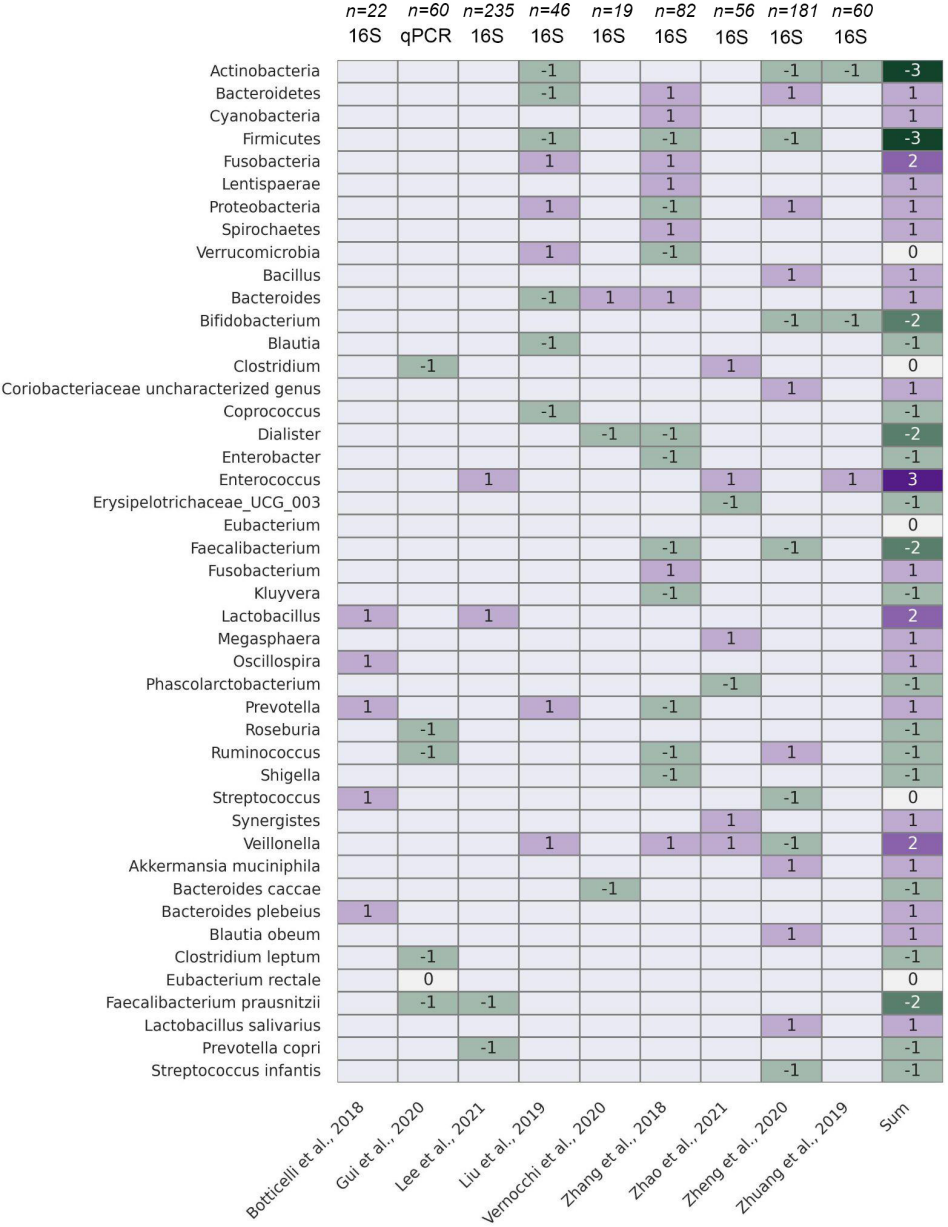
The gut microbiome and lung cancer development. Bacterial taxa at phylum, genus and species level showing significant association with lung cancer development according to case-control studies performed on real-word cohorts are displayed in a heatmap, where axis y includes taxa and axis X lists original articles (bottom) and the method of sequencing, along with total sample sizes (top). The ultimate coloumn displays the net sum of associations depicted through studies. Cells colored in magenta indicate a significant positive correlation/association, cells colored in green reflect a significant negative correlation/association between the taxa’s abundance and lung cancer incidence. Heatmap was created using Seaborn (0.13.2) from the Python Software Package.

Murine findings suggest that probiotic treatments together can restore the chemotherapy-mediated antitumor effects (341). Although, the role of *Akkermansia muciniphila* is controversial regarding murine and clinical studies, thus it needs further investigation. Cachexia, the administration of antibiotics, chemo- and immunotherapy are limiting factors in commensal microbiome studies since these strongly decrease gut microbiome diversity (4, 345). These studies underscore the urgent need for studies about the complex, significant role of the commensal gut microbiome in lung cancer development and treatment. Positive and negative effects of taxa analyzed in human case-control studies on lung cancer development are shown in **Figure 5**.

## The gut-lung axis and immunotherapy efficacy in lung cancer

In advanced-stage NSCLC immune checkpoint inhibitor (ICI) therapy is frequently administered (356, 357). Even though the ICI-treatment of NSCLC patients revolutionized treatment strategies, the limited efficacy and predictive power of the currently utilized biomarkers such as PD-L1 and tumor mutational burden is restricted. The exploration of novel non-invasive predictive biomarkers is necessary to aid patient stratification prior ICI therapy (358). The role of the intestinal microbiome as an anti-tumor response mediator came into focus in the last years in the context of different tumor types like NSCLC, melanoma, renal cell carcinoma, colorectal cancer (24, 26, 359). Although there is some similarity in the gut microorganisms linked to the response to ICI in various cancer types, this similarity is limited and cannot be accounted for by variations in sequencing techniques. Shotgun DNA sequencing and 16S rRNA sequencing are used to determine the bacterial signatures observed in the stool samples of responder and non-responder patients (360) that can further provide us information about the intra-sample variability (alpha diversity) and between-sample variability (beta diversity) of fecal samples (360, 361).

Cross-cohort analysis of NSCLC patients receiving ICI provides us controversial informations about the correlation between the microbial diversity and the therapeutic response. In terms of alpha-diversity, higher diversity was observed in responders according to Zhang et al. (362) and Lee et al. (353) after ICI treatments, and higher but not significant difference was also detected in other studies (363–365). Baseline alpha diversity was reported to be higher in future-responders (21), and it can be disturbed by antibiotic-use (27) in lung cancer patients compared to healthy individuals (365) resulting in a significantly lower level. In contrast, Dora et al. (31), and Katayama et al. (364) did not report any impact of the alpha-diversity on therapeutic response. Beta diversity showed significant differences baseline (31), and likewise post-treatment in NSCLC as well as in melanoma patients (21, 363). The aforementioned differences highlights technical divergence, and the need to interpret differently 16S rRNA (21, 27, 353, 362, 364, 365) and shotgun sequencing results (31, 363).

**Figure 6:**
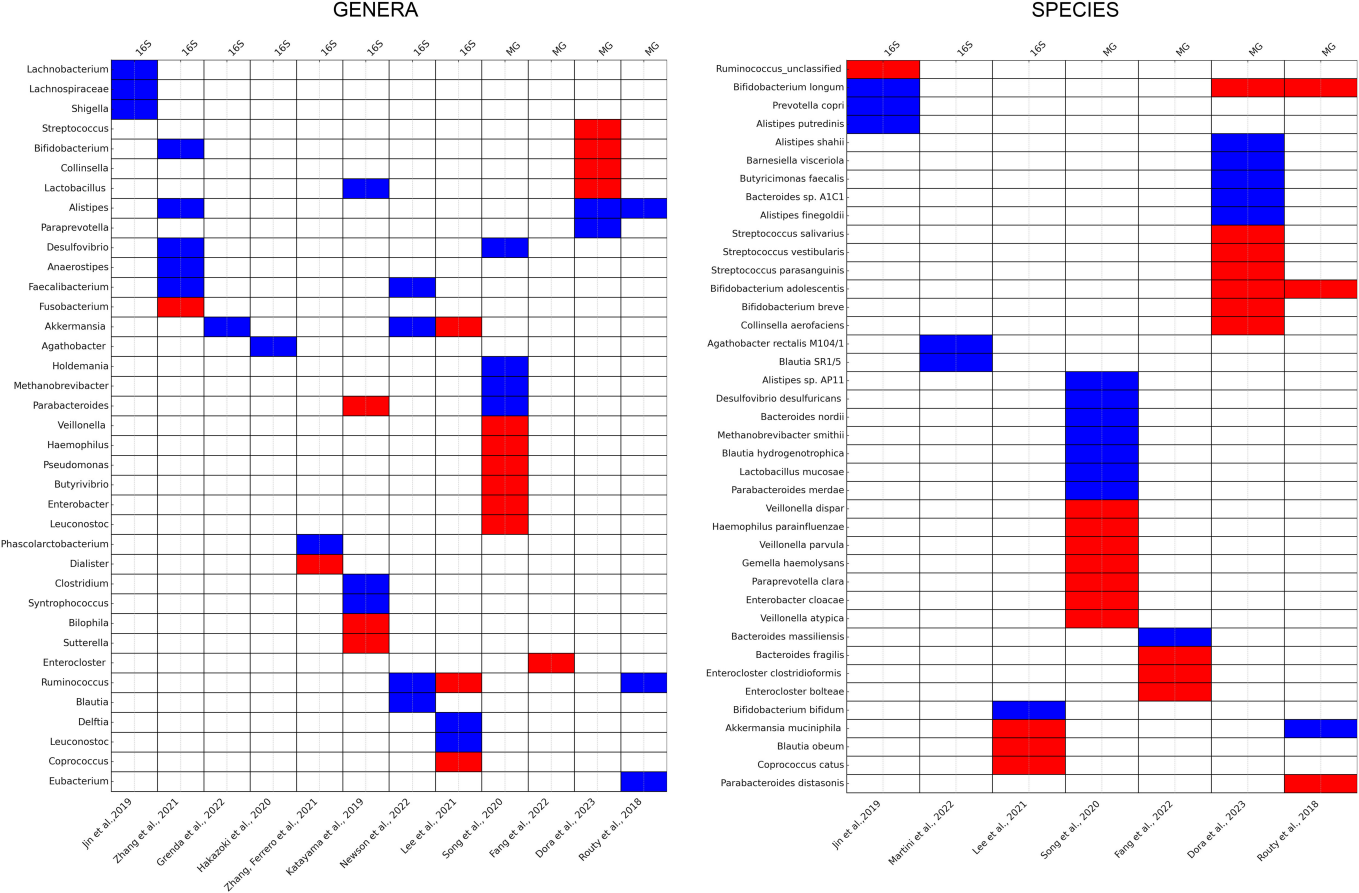
The gut microbiome and immunotherapy efficacy. Bacterial taxa at genus and species level showing significant association with response to immunotherapy (RECIST, ORR), or with progression-free survival (PFS) are displayed in a heatmap, where axis y includes taxa and axis X lists original articles (bottom) and the method of sequencing (top). Articles not assessing immunotherapy response-related patient outcomes on real-word cohorts or lacking the interpretation of differentially abundant taxa were not included. Cells colored in red indicate a significant negative correlation/association, cells colored in blue reflect a significant positive correlation/association between the taxa’s abundance and therapy response or PFS. Heatmaps were created using Seaborn (0.13.2) from the Python Software Package.

A multitude of clinical investigations have published correlations between the microbiota and the efficacy of ICIs. However, there is a lack of agreement regarding the specific taxa that are associated with treatment response in various patient groups. The lack of reliable microbial biomarkers arises from the individual variability of the microbiome influenced by host genetics, previous antibiotics use, and diet (366). Another important factor is functional diversity of the intestinal microbiome and the fact that the coexistence of host and microbiota leads to an equilibrium between the generalist functionality and diversification of individual microbes, as well as redundancy within the community (367). Geological location is also an important confounding factor through main dietary habits and host genetics. In the observed studies, most of them examined Chinese or Japanese cohorts (21, 27, 353, 362–364, 368) besides European (24, 31, 369–371) and American (365, 372). Fiber consumption is a essential dietary characteristic, which can influence the microbial composition by butyrate-producing and fibre-degrading taxa like *Rosuburia*, and representative of the *Lachnospiraceae* and *Ruminococcaceae* family (366). The presence and correlation with better ICI response of the abovementioned taxa was validated by Jin et al. (21), Hakazoki et al. (27), Chau et al. (365), Newsome et al. (372), and Routy et al. (24). High abundance of *Blautia* and *Akkermansia*, and it’s correlation with favorable outcome was reported by the most studies [*Blautia*: Martini et al. (369), Grenda et al. (370), Newsome et al. (372), Song et al. (363), *Akkermansia*: Hakazoki et al. (27), Chau et al. (365), Newsome et al. (372), Derosa et al. (373), Routy et al. (24)]. The high abundance of Firmicutes was controversial, Dora et al. (31) reported the negative predictive role of the high abundance of the microbes in contrary to Chau et al. (365). The difference can come from technical differences: Dora et al. (31) used metagenomic, while Chau et al. (365) applied 16S rRNA sequencing, and a lower number of participants. The predictive role of Parabacteroidetes was also controversial: Song et al. (363) evaluated as positive predictor in contrast to Katayama et al. (364). It’s worth to mention that the sequencing method was also different between the studies, and the uneven gender distribution in the study by Katayama et al. (364) can also have a crucial impact on the results. Two studies (31, 364) have reported a significant negative impact of *Actinobacteria*. Interestingly, the negative impact of *Bilophila, Sutterella, Parabacteroides, Bacteroides fragilis* and *Verrucomicrobia* was reported by Katayama et al. (364), Fang et al. (368), and Chau et al. (365) in contrast to a melanoma study by Matson et al. (25), however the immunological background and host factors in lung cancer and melanoma are different. The Gram-negative *Bacteroidetes* genus, *Alistipes* was found to be overrepresented in responders and patients with longer progression-free survival (PFS) by three independent studies, two of the using MG (24, 31) and one 16S rRNA sequencing (362). Dora et al. (31) also identified the genus *Streptococcus (S.)* its and multiple species, including *S. salivarius* and *S. vestibularis* to be strongly associated with decreased PFS, that is a unique finding in the field and might be explained by the utilization of the KRAKEN database instead of the more conventional Methaphlan platform. Of note, multiple hypothesis-driven studies might have focused on special taxonomic sets of bacteria, ignoring potentially significant associations with ICI-outcomes that can lead to a reporting bias. This issue can reduce the overlap between interpreted results of different studies causing an non-substantiated impression of discrepancy in the field. Establishment of comprehensive, open-access microbiome sequencing databases and better data availability may solve this problem in the long term. All observed associations are summarized in **Figure 6**.

Overall results reinforce the need for further research in this area to reveal the complex connection between the intestinal microbiome and ICI treatment outcome, and to define a clinically accessible microbiome based non-invasive predictive biomarker set. Furthermore, larger and more uniform patient cohorts are needed with prospective study designs with validation of differentially abundant taxa using cultivation or molecular studies.

## Conclusions and future perspectives

The gut-lung axis exemplifies the fundamental entwinement of the GI and respiratory systems, rooted in shared embryological origins, morphological structures, and molecular signaling pathways. Both systems develop from the primitive gut tube and are shaped by similar developmental cues involving signaling molecules like FGFs, BMPs, SHH and NOTCH receptors. These factors not only direct morphogenesis but also the functional differentiation within the gut and lungs, influencing the formation of critical structures such as the trachea, bronchi, and GI mucosa. The developmental processes are supported by neural crest cells, highlighting a shared mechanism in neural and sensory innervations, pivotal in conditions like HSCR and CCHS. However, despite the advances in understanding the morphological and molecular commonalities, current research often lacks detailed mechanistic insights into how these shared pathways explicitly influence adult disease states beyond developmental anomalies.

The local immune framework of the gut and the lungs develop from distinct anatomical sites but function in concert, mediated by the interaction of lymphoid tissues such as the GALT and the BALT. While the GALT is formed guided by intrinsic developmental programs without the necessity of microbial stimulation during embryogenesis, BALT formation requires commensal-driven noxas, both systems relying on the orchestration of lymphoid tissue inducer cells and mesenchymal lymphoid tissue organizer cells, which guide the immune landscape via cytokines and chemokines such as IL-7 and CXCL13. While foundational mechanisms of MALT formation are well-documented, the specifics of how these processes affect adult disease manifestations remain underexplored. Current studies lack depth in linking these developmental and molecular pathways directly to the pathogenesis observed in adult respiratory and GI diseases.

Regarding their microbial niches, the gut microbiome is abundant and complex, primarily aiding in metabolism and immune system development; the lung microbiome, once thought sterile, maintains a less diverse, but dynamic microbial population significantly affected by environmental air. Recent insights reveal a bidirectional communication within this axis, suggesting that imbalances in one microbiome can directly influence health outcomes in the other, where the effect seems stronger and more potent from the gut towards the lungs direction. One of the most striking examples of this interaction is the protection conferred against pneumococcal pneumonia through the gut microbiota’s enhancement of alveolar macrophage function (213) and protection against Influenza infection (374). This suggests that microbial populations in the gut can directly influence lung immunity and resistance to infections. Similarly, dysbiosis in the gut has been linked to inflammatory lung diseases such as asthma and COPD, where shifts in microbial communities appear to exacerbate pulmonary inflammation and allergic responses (375). Recent studies also revealed instances of reverse flow in biological information, from the lungs to the gut, depicted as the “lung-gut axis”, where the lung microbiota and host immune system influence gut immunity and pathologies, such as *Salmonella* infection by lung DCs (193), or expansion of ILC2 populations (201).

Human case-control studies trying to shed light on the role of the gut microbiome in lung cancer development face limitations such as interindividual varibalilty and many confounding factors affecting general health, especially smoking. Still, systematic evidence is in its emerging phase with many taxa identified as anti- or protumorigenic through interaction with the gut-lung axis, whose physiological elucidation is yet to be explored. Despite its revolutionary impact, the efficacy and predictive power of Immunotherapy biomarkers like PD-L1 and tumor mutational burden are limited, thus, novel, non-invasive predictive biomarkers are required. Recent focus has shifted to the intestinal microbiome’s role in anti-tumor responses across cancers, including NSCLC, where cross-cohort analyses showed mixed results regarding microbial diversity and ICI response. Zhang et al. (362), Jin et al. (21), and Lee et al. (353) observed higher alpha diversity in responders, whereas Dora et al. (31) and Katayama et al. (364) found no impact. Beta diversity showed significant differences pre- and post-treatment, but was also affected by prior chemotherapy-treatment, a notable confounding factor (31, 363). Numerous studies link microbiota to ICI efficacy, but specific taxa associations vary due to individual microbiome variability influenced by genetics, antibiotics, and diet (366). High *Blautia* and *Akkermansia* levels correlate with better outcomes based on cross-study evidence (369, 370, 372). While the role of *Firmicutes* shows conflicting results (31, 365), *Actinobacteria*’s negative impact and *Alistipes*’ consistent overrepresentation in responders was confirmed by multiple independent studies (24, 31, 362, 365).

Intriguingly, numerous studies present an indirect contradiction when featuring bacterial taxa associated with lung cancer/healthy state and immunotherapy-efficacy. Protumorigenic role of a certain microbe does not rule out its adjuvanticity and antigenicity during an ICI-treatment, when a robust and overboiled immune response is indispensable to reduce tumor size, even at the cost of severe ICI-related autoimmune reactions. The latter is supported by the fact that patients with ICI-toxicity exhibit increased PFS and response rates (28). Phyla are diverse taxonomic units with potentially hundreds of different species present in the gut, therefore, should not be considered as robust clinical biomarkers. Still, the unequivocal association of *Actinobacteria, Firmicutes* and *Bifidobacteria* based on case-control and murine studies with non-cancer vs lung cancer state (315, 326, 349, 350) raises the question, how these taxa can hamper ICI-efficacy based on convincing evidence (24, 31, 365)? The answer may lie within the well-known anti-inflammatory properties of these bacteria, especially *Bifidobacteria*, a common component of probiotics that might be related to their T_reg_-cell modulatory function (376). While these bacteria suppress detrimental pro-inflammatory effects in the gut that can spread carcinogenic factors locally, and via an impaired gut epithelial barrier to various organ systems, they can also suppress the excitability of an *a priori* impaired immune system of a cancer patient during the administration of ICI therapy. While several articles reported a limited, but beneficial effect of pre- and probiotics administration on ICI outcomes (376, 377), others cite controversial, or non-significant results (378, 379). Furthermore, introducing a high dose of ecologically prominent bacterium to the gut microbiome might completely alter its composition depending on the individual’s original commensal flora, resulting in unpredictable consequences. One of the still existent limitations of gut microbiome research is the disparity between the results of 16S rRNA sequencing and Metagenomic sequencing techniques. Multiple hypothesis-driven studies focus on only one- or a homogenous group of bacteria to answer a specific research question without reporting details on the whole sequencing data, creating an inherent bias.

Looking to the future, the gut-lung axis presents intriguing opportunities for novel therapeutic strategies aimed at modulating these microbiomes to treat or prevent disease. The potential to manipulate one microbiome and achieve effects in another organ system offers a promising avenue for medical research and clinical applications. Continued advancements in non-invasive sampling and more precise microbial analysis will be critical in advancing our understanding of these complex inter-organ relationships, paving the way for breakthroughs in using the microbiome to maintain health and treat disease. This evolving field promises to uncover new layers of the gut-lung axis, potentially revolutionizing our approach to a spectrum of diseases.
